# Olfactory tubercle mediates adaptive social behavior by controlling threat assessment and the expression of social threat memories during recall in male mice

**DOI:** 10.1038/s41467-026-73268-w

**Published:** 2026-05-19

**Authors:** Giulia Casarotto, Lorena Jourdain, Anastasia Gemelli, Camilla Bellone

**Affiliations:** Department of Basic Neuroscience, Medical Faculty, University of Geneva, Geneva, Switzerland

**Keywords:** Neural circuits, Long-term memory

## Abstract

The ability to dynamically assess and update threat responses based on changing environmental contexts is fundamental for survival. Here, we developed an odor-based paradigm where male mice encounter a restrained conspecific that subsequently becomes aggressive, allowing us to study how mice assess threats and update memories upon recall. Using calcium imaging, chemogenetics, and electrophysiology, we identified the olfactory tubercle (OT) as a key mediator of social threat assessment. While OT activity was not required during the initial aggressive encounter, it proved to be essential during recall, where its inhibition prevented the expression of avoidance behavior. Notably, recall induces persistent synaptic plasticity at basolateral amygdala (BLA)-to-OT synapses that persists after behavioral extinction. We identified a neuromodulatory switch in the OT: serotonin facilitates avoidance during recall, whereas its blockade triggers dopamine release and approach behavior. Our findings demonstrate that the OT orchestrates social threat assessment through synaptic plasticity and neuromodulatory control.

## Abstract (web summary)

Researchers show the olfactory tubercle mediates social threat assessment via persistent synaptic plasticity in male mice. They identify a serotonin-dopamine switch that balances avoidance and approach behaviors during the recall of social threat memories.

## Introduction

The ability to rapidly recognize potential environmental threats and react appropriately is crucial for survival across species. This dynamic threat assessment requires continuous integration of sensory inputs with prior experiences, allowing organisms to update their behavioral responses based on changing environmental contexts^1,2^. While the acute response facilitates prompt defensive actions, reconsolidation ensures that memories of threats are adaptively modified based on new information, optimizing future responses^3^. However, the neural circuits mediating this dynamic interplay between immediate threat assessment and the expression of learned threat memories remain poorly understood, particularly in naturalistic social contexts.

In rodents, paradigms such as social defeat and social fear conditioning have been used to investigate social fear^4–6^. The social defeat model involves repeated aggressive encounters where a mouse is exposed to an aggressive conspecific over multiple sessions, leading to chronic stress and long-lasting behavioral changes such as social avoidance and depressive-like symptoms^7^. Similarly, the social fear conditioning paradigm pairs a neutral social stimulus with an aversive unconditioned stimulus (often a foot shock), which upon re-exposure initiates memory reconsolidation and extinction. However, these paradigms have limitations in studying the acute neural mechanisms of threat recognition and the flexible expression of threat memories. First, the repeated nature of social defeat creates confounding effects of chronic stress that can mask acute threat processing. Second, the artificial pairing of social stimuli with foot shocks in fear conditioning fails to capture the naturalistic progression of social interactions from neutral to threatening. Third, the predictable nature of these threats prevents the study of real-time threat assessment and decision-making processes that occur during unexpected social interactions. Given these limitations, there is a critical need for paradigms that can capture the dynamic processes of threat assessment and recall-dependent changes in threat-related behavior in a more ethologically relevant context^8^, particularly those that can dissociate initial threat encoding from later recall and behavioral expression.

Although traditional mechanisms of fear response have been studied in the amygdala and hippocampus, there is growing evidence that multiple brain regions participate in these processes^9,10^. The olfactory tubercle (OT), part of the ventral striatum along with the nucleus accumbens, participates in odor-induced motivated behaviors by integrating odor identity and value^11^. Electrical stimulation of OT alters odor preference behaviour^12^ while lesion or chemogenetic silencing of the OT abolishes the attraction of female mice to male chemo signals^13,14^, suggesting its role in social approach behavior. Electrophysiological recordings further reveal that OT neurons encode the valence of odors and can discriminate between different types and magnitudes of rewards^13,15^. These findings suggest that the OT plays a broader role in processing emotionally significant stimuli, making it a promising candidate for studying threat assessment and recall-dependent expression of social threat memories.

In this study, we developed an odor-based paradigm to investigate the role of the OT in mediating threat assessment and the expression of social threat memories during recall.

Mice first encountered a restrained conspecific that later became aggressive, providing an unexpected social threat. Upon re-exposure to the aggressor in a non-threatening context (recall), we assessed how the mice updated their memory of the conspecific and adapted their behavior accordingly. Our findings reveal a complex mechanism by which the OT orchestrates social threat processing. OT activity was critical during recall, where its inhibition prevented the expression of social avoidance at both acute (1 h) and sustained (24 h) recall time points. We observed a decrease in the strength of the synapses at the basolateral amygdala (BLA) to OT inputs after recall that persisted after the extinction of avoidance behavior. Building on this finding, we investigated the neuromodulatory mechanisms driving avoidance behavior and found that serotonin release in the OT during recall facilitated avoidance, while its blockade triggered dopamine release and promoted approach behavior. Together, these results reveal how the OT, through precise neuromodulatory control and synaptic plasticity, mediates the dynamic assessment and the recall-dependent expression of social threat memories.

## Results

### Social threat avoidance is stimulus-specific and not generalizable

**Fig. 1:**
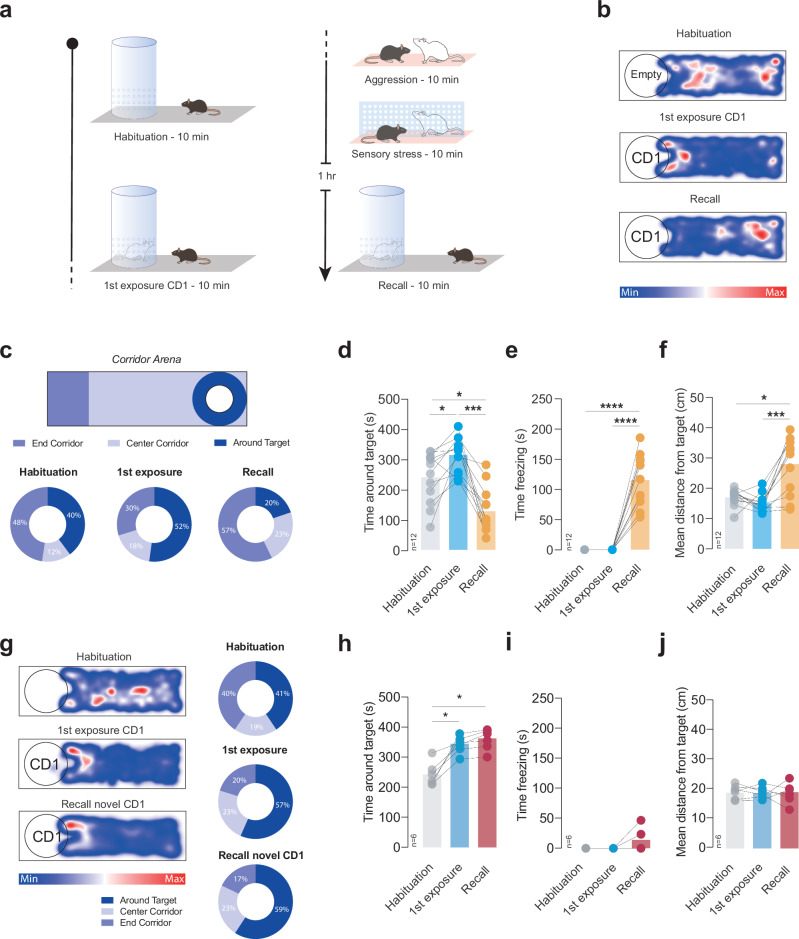
Social threat avoidance is stimulus-specific and not generalizable. **a** Schematic representation of the behavioral task. **b** Heatmap reporting the mean occupancy of the batch of mice during the different phases of the test. **c** Top: Schematic representation of the experimental arena partitioned into three zones: End Corridor (medium blue), Center Corridor (light blue), and Around Target (dark blue). Bottom: Pie chart representing the mean percentage of time spent by mice in each zone during three distinct behavioral phases. **d** Time around enclosure containing stimulus (RM one-way ANOVA: F1.584, 17.42 = 19.48, *p* = 0.000081, followed by Bonferroni multiple comparison post hoc test). **e** Time freezing during the test (RM one-way ANOVA: F1.000, 10.00 = 77.58, *p* = 0.000005, followed by Bonferroni multiple comparison post hoc test). **f** Mean distance between nose point of the experimental mouse and the center of the target during the test (RM one-way ANOVA: F1.204, 13.25 = 16.62, *p* = 0.0008, followed by Bonferroni multiple comparison post hoc test). **g** Left: Heatmap reporting the mean occupancy of the batch of mice during the different phases of the test. Right: Pie chart representing the mean percentage of time spent by mice in each zone during three distinct behavioral phases. **h** Time around enclosure containing stimulus (RM one-way ANOVA: F1.082, 4.328 = 19.99, *p* = 0.0088, followed by Bonferroni multiple comparison post hoc test). **i** Time freezing during the test (RM one-way ANOVA: F1.000, 5.000 = 2.150, *p* = 0.2025, followed by Bonferroni multiple comparison post hoc test). **j** Mean distance between nose point of the experimental mouse and the center of the target during the test (RM one-way ANOVA: F1.199, 5.99 = 0.02886, *p* = 0.9062, followed by Bonferroni multiple comparison post hoc test). n indicates the number of mice. All the data are shown as the mean ± s.e.m. as error bars. Source data are provided as a Source Data file.

To investigate the acute neural mechanisms of social threat recognition and the expression of social threat memories, we developed a behavioral paradigm in which mice first encounter a restrained adult CD1 and then experience a brief episode of direct aggression with the same animal (Fig. 1a). This sequential procedure allows us to study how mice assess and adapt to a recently experienced social threat in a controlled, ethologically inspired context.

The experiment began with a 10-minute habituation period in a corridor-like arena, during which the experimental mice were exposed to an empty opaque dark plastic enclosure with small openings. Following habituation, we introduced a CD1 mouse into the enclosure for a 10 min session (first exposure to CD1). During this initial exposure, the experimental mice approached and investigated the enclosure, indicating that the presence of the CD1 mouse promotes approach behavior (Fig. 1b). It should be noted that the specific design of the enclosure enhances olfactory responses while minimizing visual and tactile interactions^16^.

Immediately after the first exposure, the experimental mice were placed directly into the home cage of the CD1 mouse for a 10-minute free interaction period (aggression). This setup allowed for unrestricted social interaction between the mice, enabling them to engage in natural behaviors and experience direct social cues from the CD1 mouse, including aggressive behaviors. Subsequently, the mice were separated from the CD1 mouse by a Plexiglas barrier and remained in the home cage for an additional 10 min to increase sensory stress.

After returning to their home cages for one hour, the experimental mice were reintroduced into the arena for a second exposure to the same CD1 mouse confined within the opaque enclosure (recall exposure to CD1). During this recall exposure, mice displayed a clear avoidance response characterized by a significant decrease in exploration time near the CD1 enclosure (Fig. 1c, d). This reduction in exploration time was accompanied by increased freezing behavior, a greater mean distance from the enclosure, and a decreased number of entries into the target zone (Fig. 1e, f). Furthermore, an analysis of the transition probabilities between grooming, rearing, and investigation of the enclosure across sessions reveals a shift in the dynamics of these behaviors (Supplementary Fig. 1a). These findings suggest that mice exhibit avoidance of the CD1 mouse upon recalling the initial interaction.

To ensure that the observed avoidance behavior was not due to habituation to the task itself, we replicated the experiment using a juvenile conspecific as the stimulus (Supplementary Fig. 1b). Under these conditions, we observed no significant differences in interaction time or freezing behavior between the first and recall exposures (Supplementary Fig. 1c–h). This lack of an adaptive avoidance response indicates that the avoidance behavior is specifically triggered by the prior aggressive experience with the adult CD1 mouse, rather than by general habituation to the experimental setup.

We also wanted to determine whether the avoidance behavior was specific to the initial aggressor. We introduced a different CD1 mouse during the recall exposure (Fig. 1g) and we observed that mice did not exhibit avoidance behavior toward the unfamiliar CD1 mouse (Fig. 1h–j). This specificity indicates that social avoidance is not generalizable but rather results from the negative association formed during the initial encounter with the specific aggressor. Finally, we assessed the context dependency of the avoidance behavior. To do so, we performed the same behavioral experiment changing the context during the recall phase (Supplementary Fig. 1i). We found that mice continued to avoid the CD1 aggressor with which they had previously experienced direct aggression and showed increased freezing, despite the change in context (Supplementary Fig. 1j, k). These findings demonstrate that the observed social avoidance is highly specific to the identity of the social threat and is independent of the environmental context.

### Persistence and dynamic of social threat memories over time

**Fig. 2:**
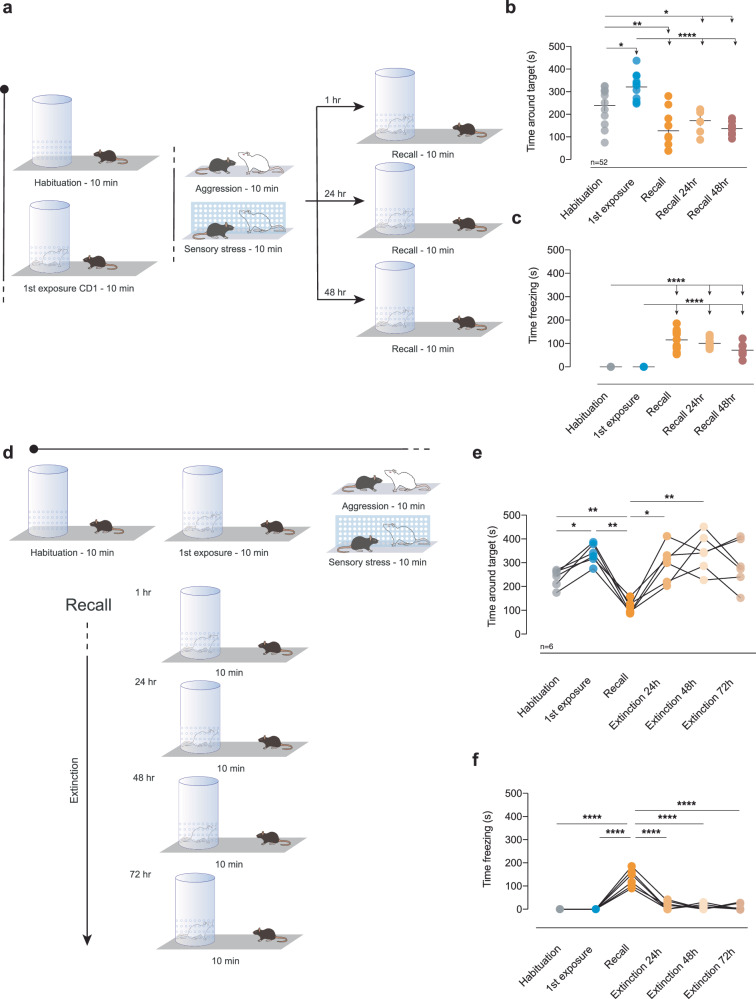
Persistence and dynamic of social threat memories over time. **a** Schematic of the experimental timeline. **b** Time around enclosure containing stimulus (RM one-way ANOVA: F4, 47 = 17.95, *p* = 0.000000052, followed by Bonferroni multiple comparison post hoc test). **c** Time freezing during the test (RM one-way ANOVA: F4, 44 = 49.53, *p* < 0.0000000001, followed by Bonferroni multiple comparison post hoc test). **d** Schematic of the experimental timeline. **e** Time around enclosure containing stimulus (RM one-way ANOVA: F2.203, 11.01 = 13.02, *p* = 0.0011, followed by. Bonferroni multiple comparison post hoc test). **f** Time freezing during the test (RM one-way ANOVA: F5, 30 = 36.15, *p* = 0.000000000008, followed by Bonferroni multiple comparison post hoc test). n indicates the number of mice. All the data are shown as the mean ± s.e.m. as error bars. Source data are provided as a Source Data file.

To investigate whether social avoidance behavior persists over extended periods and remains susceptible to extinction learning, we examined the temporal stability of social threat memories. Mice were subjected to our behavioral paradigm (Fig. 1a), but instead of testing recall after one hour, we returned them to their home cages and assessed social avoidance at 24 or 48 h post-aggression (Fig. 2a). Analysis of behavioral metrics revealed that mice exhibited robust maintenance of social avoidance at these extended time points, displaying a significant increase in freezing behavior and a sustained reduction in interaction time with the CD1-containing enclosure—comparable to the responses observed at the one-hour recall time point (Fig. 2b, c). These behavioral adaptations demonstrated that social threat memories undergo stable consolidation and persist across extended periods.

We next examined whether consolidated threat memories could be modified through extinction learning (Fig. 2d). Following initial recall testing, all mice underwent a multi-day extinction protocol consisting of extended daily re-exposures to the aggressor CD1 mouse over a 72 h period. Behavioral analysis revealed progressive extinction of both avoidance and freezing behaviors across these sessions (Fig. 2e, f). These data demonstrate that while social threat memories persist stably over time, they can be modified through repeated non-aggressive re-exposures to the aggressor confined in the enclosure.

### OT activity is necessary for social assessment during the recall

**Fig. 3:**
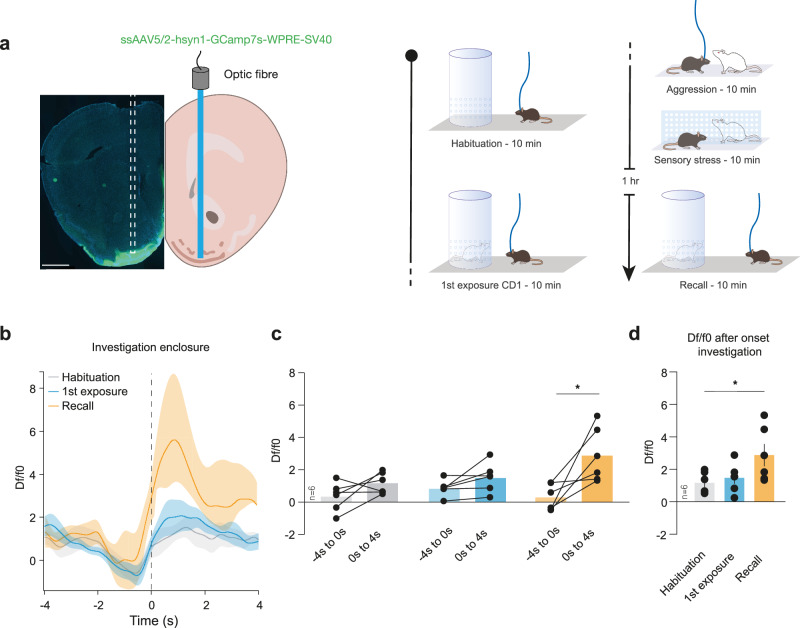
OT neurons exhibit increased activity during social threat assessment. **a** Left: Representative picture and schematic of the viral injection and optic fiber implantation (scale bar: 750 µm). Similar viral expression and optic fiber location were observed in all the mice that performed the experiment described in (**b**–**d**). Right: Schematic of the experimental timeline. **b** Mean DF/F0 signal ±4 s around investigation of the enclosure onset (indicated by dashed line, 0 s) during habituation, 1^st^ exposure and recall. **c** Left: Quantification of DF/F0 difference before and after investigation of the enclosure onset during habituation (paired t-test two-sided: t1.777 = 5, *p* = 0.1357). Middle: Quantification of DF/F0 difference before and after investigation of the enclosure onset during 1^st^ exposure to CD1 (paired t-test two-sided: t2.061 = 5, *p* = 0.0943). Right: Quantification of DF/F0 difference before and after investigation of the enclosure onset during recall (paired t-test two-sided: t3.201 = 5, *p* = 0.0240). **d** Comparison of DF/F0 after investigation of the enclosure onset for the three different test phases (one-way ANOVA: F = 3.833, p = 0.0452, followed by Bonferroni multiple comparison post hoc test). n indicates the number of mice. All the data are shown as the mean ± s.e.m. as error bars. Source data are provided as a Source Data file.

Given the essential role of olfactory cues in our social threat paradigm, we hypothesize that the olfactory tubercle (OT), a ventral striatal structure integral to odor-guided motivated behaviors^11^, plays a critical role in social threat recognition and behavioral adaptation during recall. To test this hypothesis, we injected an adeno-associated virus (AAV) expressing GCaMP7s and implanted an optic fiber in the OT to measure calcium transients during the different phases of our behavioral task (Fig. 3a, Supplementary Fig. 2a, b). This approach allows us to monitor real-time neural activity in the OT as mice assess and adapt to social threats, providing insights into the neural mechanisms underlying adaptive social behaviors. Calcium transient analysis revealed phase-specific activation patterns in the OT. During habituation and first exposure, mice showed no significant modulation of OT calcium transients at the onset of enclosure investigation (Fig. 3b, c). In contrast, significant changes in neural activity were detected during the recall phase, where OT activity was significantly elevated following investigation onset compared to both habituation and first exposure (Fig. 3d).

To further characterize OT activity patterns, we aligned calcium signals to specific behaviors, including rearing, grooming, and freezing across all experimental phases. These behaviors did not evoke consistent OT responses during habituation, first exposure, aggression and recall phases (Supplementary Fig. 2c–p). These findings identify the OT as a key node in the neural circuit mediating social threat recognition.

**Fig. 4:**
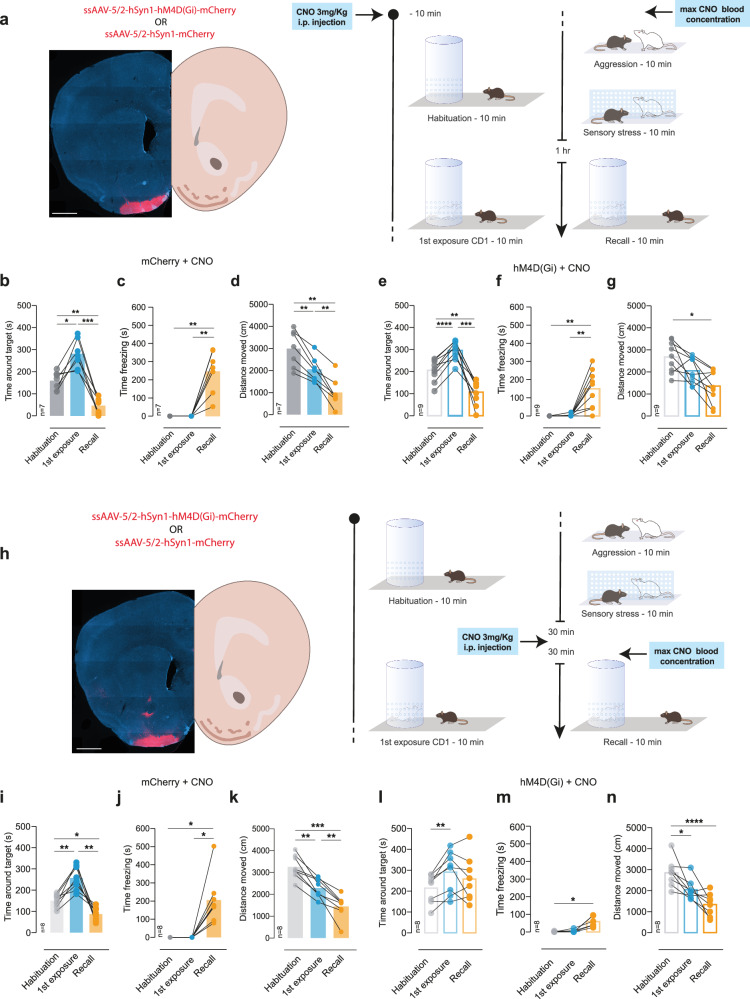
Chemogenetic inhibition of the OT abolishes avoidance during recall. **a** Left: Representative picture and schematic of the injection (scale bar: 750 µm). Similar viral expression was observed in all the mice that performed the experiment described in (**b**–**g**, **i**–**n**). Right: Schematic of the experimental timeline. **b** Time around enclosure (RM one-way ANOVA: F1.587, 9.521 = 40.44, *p* = 0.000037, followed by Bonferroni multiple comparison post-hoc test). **c** Time freezing (RM one-way ANOVA: F1.000, 6.000 = 30.79, *p* = 0.0014, followed by Bonferroni multiple comparison post-hoc test). **d** Distance moved (RM one-way ANOVA: F1.239, 7.432 = 43.35, *p* = 0.0002, followed by Bonferroni multiple comparison post-hoc test). **e** Time around enclosure (RM one-way ANOVA: F1.313, 10.50 = 44.21, *p* = 0.000022, followed by Bonferroni multiple comparison post-hoc test). **f** Time freezing (RM one-way ANOVA: F1.006, 8.0048 = 17.49, *p* = 0.0030, followed by Bonferroni multiple comparison post-hoc test). **g** Distance moved (RM one-way ANOVA: F1.791, 14.33 = 9.844, *p* = 0.0025, followed by Bonferroni multiple comparison post-hoc test). **h** Left: Representative picture and schematic of the viral injection (scale bar: 750 µm). Right: Schematic of the experimental timeline. **i** Time around enclosure (RM one-way ANOVA: F1.196, 8.372 = 30.50, *p* = 0.0003, followed by Bonferroni multiple comparison post-hoc test). **j** Time freezing (RM one-way ANOVA: F1.000, 7.000 = 12.78, *p* = 0.009, followed by Bonferroni multiple comparison post-hoc test). **k** Distance moved (RM one-way ANOVA: F1.247, 8.732 = 38.55, *p* = 0.0001, followed by Bonferroni multiple comparison post-hoc test). **l** Time around enclosure (RM one-way ANOVA: F1.464, 10.25 = 4.625, *p* = 0.0456, followed by Bonferroni multiple comparison post-hoc test). **m** Time freezing (RM one-way ANOVA: F1.077, 7.537 = 9.629, *p* = 0.0148, followed by Bonferroni multiple comparison post hoc test). **n** Distance moved (RM one-way ANOVA: F1.922, 13.45 = 17.04, *p* = 0.0002, followed by Bonferroni multiple comparison post-hoc test). n indicates the number of mice. All the data are shown as the mean ± s.e.m. as error bars. Source data are provided as a Source Data file.

To elucidate the causal relationship between OT activity and observed behaviors, we adopted a chemogenetic strategy. We injected either ssAAV-5/2-hSyn1-hM4D(Gi)-mCherry for OT inhibition or ssAAV-5/2-hSyn1-mCherry as a control into the OT of different mice groups (Fig. 4a). Clozapine N-oxide (CNO) was administered intraperitoneally (i.p.) 30 min before either the aggression period or the recall (Fig. 4a, h). We found that OT inhibition during the aggression phase does not change the behavioral outcome of the recall phase compared to control conditions (Fig. 4b–g). In contrast, when OT activity was blocked during the recall, the difference in interaction time between the first encounter and recall was abolished (Fig. 4i–n). Interestingly, the enhanced freezing response persisted (Fig. 4m).

To further validate that OT activity is specifically required during recall, we blocked OT function 24 h after the aggression experience, at the time when the recall phase normally induces a decrease in time spent around the target (Supplementary Fig. 3a). Consistent with our previous findings, control mice displayed the expected avoidance during recall, whereas CNO administration 30 minutes prior to recall prevented this avoidance response (Supplementary Fig. 3b–e).

Because the OT and nucleus accumbens (NAc) are both components of the ventral striatum, we next examined whether the recall effect reflected a broader requirement for ventral striatal function. Chemogenetic inhibition of the NAc 30 min before recall had no effect: mice displayedavoidance, comparable to controls (Supplementary Fig. 3f, g). This control demonstrates that the behavioral deficit is not due to perturbing ventral striatal circuits generally but reflects a specific requirement for OT activity during recall.

Finally, we asked whether the requirement for OT activity during recall was specific to social threat processing or reflected a more general role in aversion or avoidance. To test this, we examined responses to the innate predator odor trimethylthiazoline (TMT). Chemogenetic inhibition of the OT 30 min before TMT exposure did not alter avoidance behavior: mice displayed robust aversion to TMT indistinguishable from controls (Supplementary Fig. 3h–j). This result indicates that OT activity is not required for innate threat responses and suggests that its contribution is specific to experience-dependent social threat evaluation, rather than mediating avoidance behaviors per se.

Together, these findings demonstrate that OT activity during recall is crucial for translating prior social experiences into appropriate defensive behaviors, suggesting a key role for this structure in the retrieval and behavioral expression of social threat memories.

### Recall-dependent synaptic plasticity at BLA to OT synapses

**Fig. 5:**
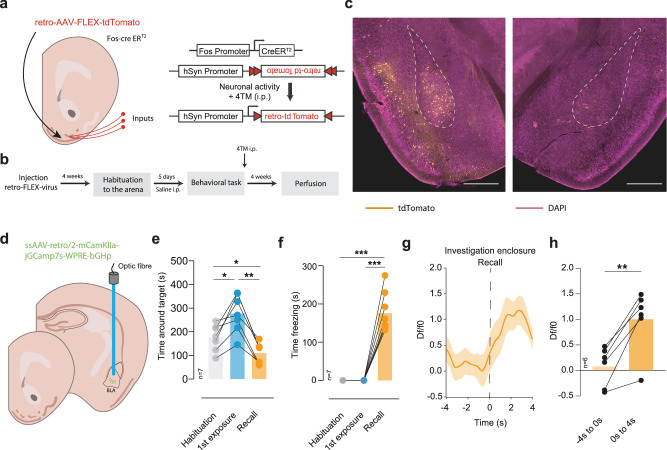
BLA to OT pathway is active during threat assessment. **a** Left: Schematic of the viral injection (*n* = 3 mice, this experiment was reproduced three times). Right: Schematic of the transgenic construct after activation by 4-OH tamoxifen injection. **b** Schematic of the experimental timeline. **c** Representative pictures of active neuronal inputs from basolateral amygdala (right: ipsilateral to the injection site, left: contralateral to the injection side, (scale bar: 500 µm). **d** Schematic of the viral injection and optic fiber implantation. **e** Time around enclosure containing stimulus (RM one-way ANOVA: F1.196, 8.372 = 30.50, *p* = 0.0003, followed by Bonferroni multiple comparison son post hoc test). **f** Time freezing during the test (RM one-way ANOVA: F1.000, 6.000 = 80.42, *p* = 0.000107, followed by Bonferroni multiple comparison post hoc test). **g** Mean DF/F0 signal ±4 s around investigation enclosure onset (indicated by dashed line, 0 s) during threat recall. **h** Quantification of DF/F0 difference before and after investigation enclosure onset during recall (paired t-test two-sided: t5.380 = 5, *p* = 0.0030). n indicates the number of mice. All the data are shown as the mean ± s.e.m. as error bars. Source data are provided as a Source Data file.

To identify the neural circuits involved in memory retrieval and the recall-dependent expression of social threat memories, we employed tracing and in vivo calcium imaging to pinpoint key brain regions activating the OT during the recall phase. In Fos-Cre-ERT2 mice, we injected a retrograde AAV-FLEX-tdTomato virus into the OT to label neurons projecting to the OT that are active during recall (Fig. 5a). Four weeks post-injection, mice underwent the behavioral paradigm, followed by intraperitoneal administration of tamoxifen to induce Cre recombinase activity in the neurons engaged during recall (Fig. 5b).

Post-behavioral assessment revealed significant activation across multiple brain regions involved in social behavior and threat processing. Notably, the basolateral amygdala (BLA) exhibited the most robust activation among the identified regions (Fig. 5c, Supplementary Fig. 4a), suggesting an important role for BLA projections in modulating OT activity during threat assessment.

To determine the cellular identity of the BLA projections to the OT, we used a bicolor retrograde viral strategy. Specifically, we injected AAVrg-ef1a-DIO-EGFP-tdTomato into the OT of GAD2-Cre mice (Supplementary Fig. 4b). This approach allowed for selective labeling of GABAergic (inhibitory) and non-GABAergic (putatively glutamatergic) projections from the BLA. Immunohistochemical analysis revealed that the majority of BLA projections to the OT did not co-express the inhibitory marker GAD2, indicating that these projections are predominantly excitatory (Supplementary Fig. 4c).

To directly examine the functional dynamics of this pathway, we investigated the activity patterns of glutamatergic BLA neurons that project to the OT. We used a projection-specific approach by injecting a retrograde GCaMP7s-expressing virus (ssAAV-retro/2-mCamKIIa-jGCaMP7s-WPRE-bGHp) into the OT and implanting an optic fiber in the BLA (Fig. 5d, e). Analysis of calcium transients revealed distinct temporal patterns of BLA-OT projection neurons across behavioral phases. When aligning neural activity to different behavioral events (Supplementary Fig. 4d–o), we observed that these glutamatergic neurons displayed robust activation during non-threat-related behaviors, such as grooming, independent of the experimental phase (Supplementary Fig. 4f, g, j, k, n, o). However, during the recall phase, BLA-OT neurons exhibited an additional, context-specific pattern of activation when mice were exposed to the previously aggressive CD1 mouse (Fig. 5 g, h). This threat-related activation was particularly pronounced during approaches to the CD1-containing enclosure, suggesting that the BLA plays a crucial role in modulating OT activity in response to socially relevant threats.

**Fig. 6:**
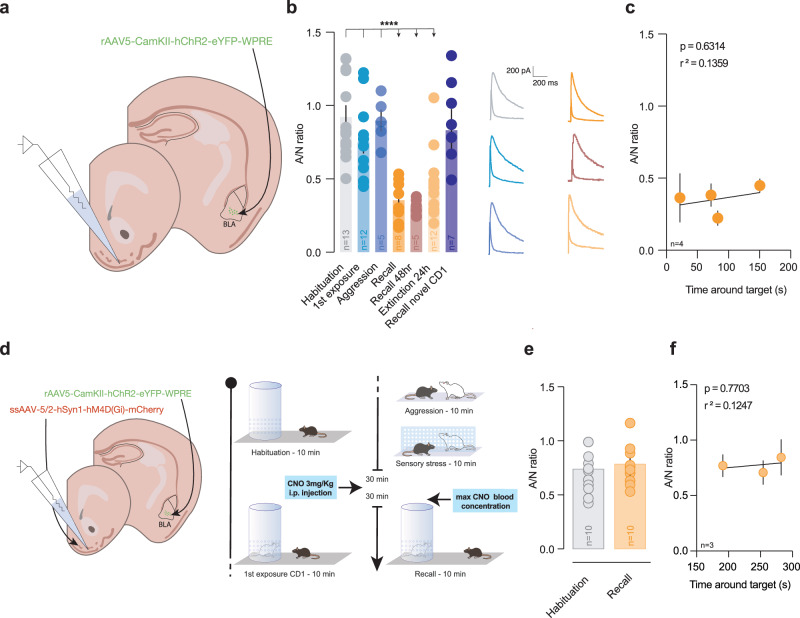
Threat recall induces synaptic plasticity at BLA to OT synapses. **a** Schematic of the viral injection. **b** Left: Bar graph representing AMPAR/NMDAR ratio recorded after different phases of the test (Ordinary one-way ANOVA: F(6, 53) = 9.597, *p* = 0.00000039). Right: Representative traces of AMPAR/NMDAR currents. **c** Correlation between AMPAR/NMDAR ratio and time around target for the recall phase for each mouse (Pearson correlation: R^2^ = 0.1359, *p* = 0.6314). **d** Left: Schematic of the viral injections. Right: Schematic of the experimental timeline. **e** Bar graph representing. AMPAR/NMDAR ratio recorded after different phases of the test (unpaired t-test two-sided: t0.4449 = 18. *p* = 0.3029). **f** Correlation between AMPAR/NMDAR ratio and time around target for the recall phase for each mouse Pearson correlation: R^2^ = 0.1247, *p* = 0.7703). n indicates the number of cells in (**b**, **e**) and the number of animals in (**c**, **f**). All the data are shown as the mean ± s.e.m. as error bars. Source data are provided as a Source Data file.

Building on the observed activation of the BLA-OT pathway during recall, we hypothesized that neural activity in this circuit facilitates synaptic plasticity within the BLA-OT pathway, potentially serving as a cellular mechanism for behavioral adaptation. To test this hypothesis, we targeted BLA neurons with a virus expressing Channelrhodopsin-2 (ChR2). After a three-week expression period, mice were divided into cohorts, each exposed to different behavioral test sessions. Twenty-four hours after each phase, we prepared brain slices containing the OT for whole-cell patch-clamp recordings (Fig. 6a).

Electrophysiological recordings revealed a significant decrease in the AMPA/NMDA ratio of excitatory postsynaptic currents (EPSCs) specifically after the recall session (Fig. 6b), but no changes in RI (Supplementary Fig. 5a). Notably, this synaptic modification persisted even after extinction learning (Fig. 6b), suggesting that extinction modifies the original threat memory trace rather than erasing it. This persistent synaptic change indicates that the BLA-OT pathway undergoes long-lasting plasticity associated with changes in the expression of social threat memories. Consistent with a recall-triggered, pathway-specific process rather than a simple reflection of behavioral variability, the AMPA/NMDA ratio did not correlate with the time spent around the target (Fig. 6c). To determine the pathway specificity of these synaptic changes, we examined another major input to the OT by targeting the olfactory bulb (OB) with ChR2. Whole-cell patch-clamp recordings of OT neurons 24 h after recall revealed no changes in synaptic responses following OB stimulation (Supplementary Fig. 5b, c), indicating that recall-induced plasticity is specific to the BLA-OT pathway.

Finally, to test for a causal link between BLA-OT activity, social avoidance behavior, and synaptic plasticity, we combined chemogenetic inhibition of the OT using hM4Di with optogenetic activation of BLA inputs (ChR2) (Fig. 6d). Chemogenetic suppression of OT activity prior to the recall exposure prevented the expression of social avoidance behavior and the decrease in the AMPA/NMDA ratio typically observed 24 h after recall (Fig. 6e). Analysis of this cohort showed that while both the behavior and the plasticity were successfully blocked, there was no correlation between the AMPA/NMDA ratio and the time spent around the enclosure (Fig. 6f). Together, these results demonstrate that activation of the BLA–OT circuit during recall is required for both behavioral adaptation and pathway-specific long-lasting synaptic plasticity.

### DA and 5HT balance in social approach/avoidance behavior

Having established that the OT is required during recall and that BLA → OT inputs undergo recall-dependent plasticity, we next sought to identify the neuromodulatory mechanisms that regulate OT output during threat assessment. Because synaptic plasticity provides a substrate for storing updated threat information while neuromodulators shape moment-to-moment behavioral responses, we reasoned that monoaminergic systems might act on the OT to gate the expression of avoidance or approach following memory retrieval. In particular, dopamine and serotonin—both of which densely innervate the OT—are strong candidates to dynamically tune OT processing during recall^16–19^. We therefore examined how DA and 5-HT signals interact with the OT circuitry during social threat evaluation.

**Fig. 7:**
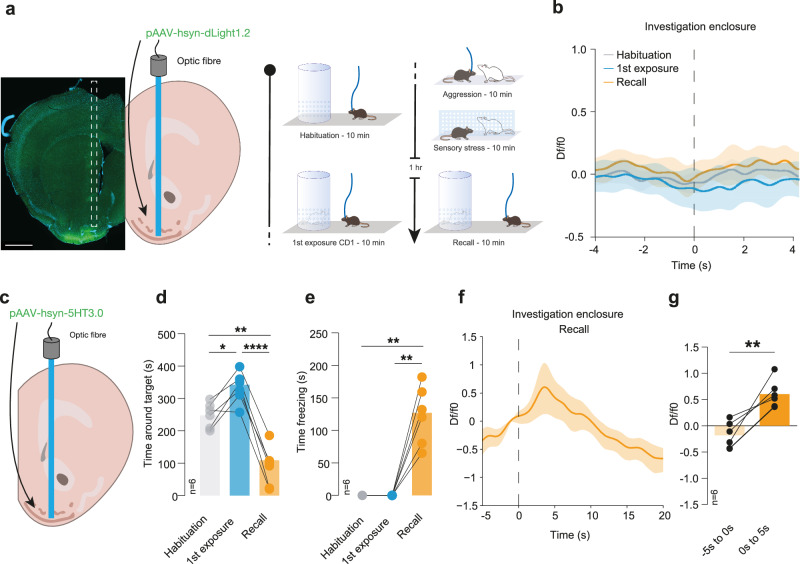
DA and 5HT balance in social approach/avoidance behavior. **a** Left: Representative picture and schematic representation of the viral injection (scale bar: 750 µm). Similar viral expression and optic fiber location were observed in all the mice that performed the experiment described in (**b**, **d**–**g**). Right: Schematic of the experimental timeline. **b** Mean DF/F0 signal ±4 s around investigating enclosure onset (indicated by dashed line, 0 s) during habituation, 1^st^ exposure and recall. **c** Schematic representation of the viral injection. Right: Schematic of the experimental timeline. **d** Time around enclosure containing stimulus (RM one-way ANOVA: F1.834, 9.17 = 75.37, p = 0.000002, followed by Bonferroni multiple comparison post hoc test). **e** Time freezing during the test (RM one-way ANOVA: F1.000, 5.000 = 45.11, *p* = 0.0011, followed by Bonferroni multiple comparison post hoc test). **f** Mean DF/F0 signal ±4 s around sniffing enclosure initiation (indicated by dashed line, 0 s) during threat recall. **g** Quantification of DF/F0 difference before and after sniffing enclosure during the threat recall (paired t-test: t5.506 = 4, *p* = 0.0027). n indicates the number of mice. All the data are shown as the mean ± s.e.m. as error bars. Source data are provided as a Source Data file.

To directly measure DA dynamics, we expressed a dopamine sensor (pAAV-hSyn-dLight1.2) in the OT and implanted an optic fiber in the same region (Fig. 7a, Supplementary Fig. 6a). After a three-week expression period, mice were subjected to our behavioral protocol. Surprisingly, we did not observe dopamine signals time-locked to the enclosure proximity in the OT during any phase of the behavioral task (Fig. 7b, Supplementary Fig. 6b–h). Interestingly, we observed DA signals in the OT when the mouse explored an unfamiliar juvenile conspecific after the CD1 aggressive experience (Supplementary Fig. 6i–k), indicating that, as expected, DA release in the OT is engaged during positive, rewarding social interactions.

This finding prompted us to explore the potential involvement of 5HT in regulating social avoidance behavior. We first confirmed the presence of a direct serotonergic projection to the OT by injecting a Cre-dependent virus (ssAAV-9/2-hsyn1-dlox-tdTomato(rev)-dlox-WPRE-bGHp(A)) into the dorsal raphe (DR) of SERT-Cre mice; histological analysis revealed a dense plexus of serotonergic fibers within the OT (Supplementary Fig. 7a). To probe 5-HT release, we injected the 5-HT sensor into the OT and implanted an optic fiber in the same region (Fig. 7c, Supplementary Fig. 6l). Our fiber photometry recordings revealed 5-HT release within the OT exclusively during the recall phase when mice approached the encounter containing the potential threat (Fig. 7d–g, Supplementary Fig. 6m–o).

These results revealed a dissociation between neuromodulatory signals in the OT, where DA phasic release is not observed across all behavioral phases. In contrast, 5-HT release exhibited a time-specific activation pattern coinciding with threat assessment during recall, suggesting selective recruitment of 5-HT signaling in social threat processing.

**Fig. 8:**
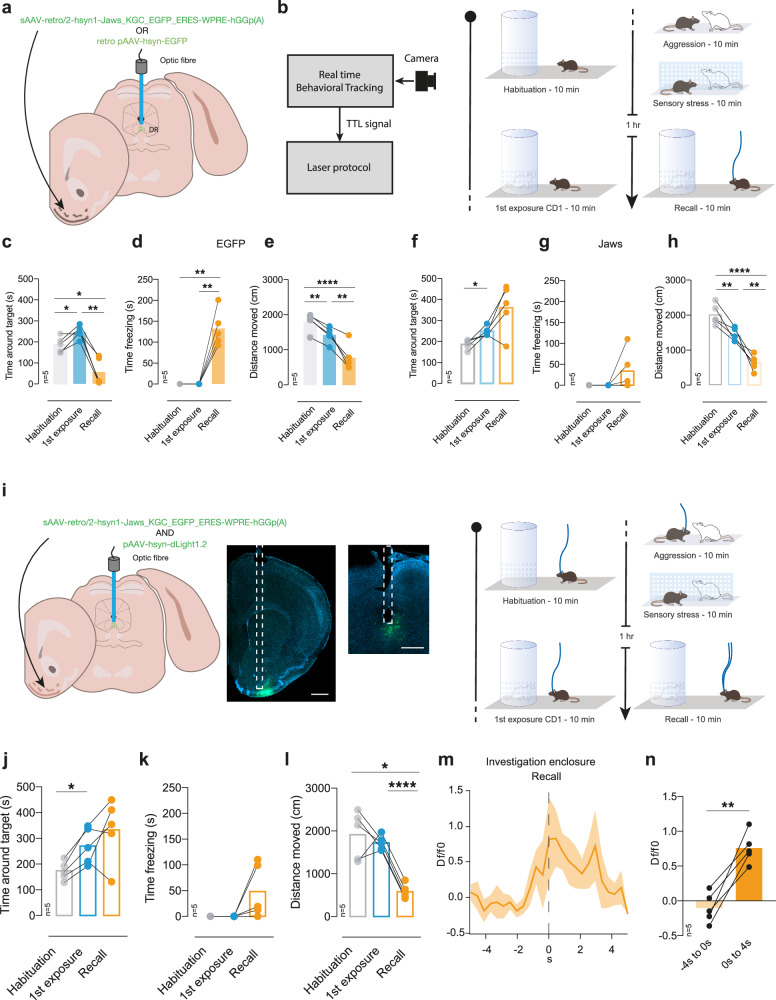
5HT inhibition promotes approach disinhibiting DA release in the OT. **a** Schematic representation of the viral injection. Similar viral expression and optic fiber location were observed in all the mice that performed the experiment described in (**j**–**n**). **b** Left: Schematic of the closed loop stimulation system. Right: Schematic of the experimental timeline. **c** Time around enclosure containing stimulus (RM one-way ANOVA: F1.410, 5.640 = 43.15, *p* = 0.0005, followed by Bonferroni multiple comparison post-hoc test). **d** Time freezing during the test (RM one-way ANOVA: F1.000, 4.000 = 47.07, *p* = 0.0024, followed by Bonferroni multiple comparison post-hoc test). **e** Distance moved in apparatus (RM one-way ANOVA: F1.048, 4.193 = 35.84, *p* = 0.0033, followed by Bonferroni multiple comparison post-hoc test). **f** Time around enclosure containing stimulus (RM one-way ANOVA: F1.026, 4.103 = 8.133, *p* = 0.0447, followed by Bonferroni multiple comparison post-hoc test). **g** Time freezing during the test (RM one-way ANOVA: F1.000, 4.000 = 2.58, *p* = 0.1833, followed by Bonferroni multiple comparison post-hoc test). **h** Distance moved in apparatus (RM one-way ANOVA: F1.644, 6.575 = 37.27, *p* = 0.0003, followed by Bonferroni multiple comparison post-hoc test). **i** Left: Schematic representation of the viral injection and representative pictures (left scale bar: 750 µm, right scale bar: 500 µm). Right: Schematic of the experimental timeline. **j** Time around enclosure containing stimulus (RM one-way ANOVA: F1.159, 4.636 = 6.368, *p* = 0.0546, followed by Bonferroni multiple comparison post-hoc test). **k** Time freezing during the test (RM one-way ANOVA: F1.000, 4.000 = 4.273, *p* = 0.1076, followed by Bonferroni multiple comparison post-hoc test). **l** Distance moved in apparatus (RM one-way ANOVA: F1.003, 4.013 = 20.07, *p* = 0.0109, followed by Bonferroni multiple comparison post-hoc test). **m** Mean DF/F0 signal ±4 s around investigating enclosure onset (indicated by dashed line, 0 s) during threat recognition. **n** Quantification of DF/F0 difference before and after investigating the enclosure onset during recall (paired t-test two-sided: t6.204 = 4, *p* = 0.0034). n indicates the number of mice. All the data are shown as the mean ± s.e.m. as error bars. Source data are provided as a Source Data file.

To directly test whether the 5-HT release is necessary for social avoidance behavior during recall, we optogenetically inhibited putative 5-HT neurons projecting from the DR to the OT. This was achieved by targeting the OT either with a retrograde virus expressing Jaws (sAAV-retro/2-hSyn1-Jaws_KGC_EGFP_ERES-WPRE-hGGp(A)) or, as a control, a retrograde virus expressing EGFP (retro pAAV-syn-EGFP) and implanting an optic fiber in the DR (Fig. 8a, Supplementary Fig. 7b). After a three-week expression period, we performed the behavioral task while optogenetically inhibiting DR-OT serotonin neurons (Fig. 8b). Inhibition was selectively applied during the recall exposure, triggered in a closed-loop manner when mice were in proximity to the CD1 enclosure (See Methods). Optogenetic inhibition of this pathway abolished the social avoidance behavior typically observed in control animals (Fig. 8c–h). Strikingly, inhibition not only blocked avoidance behavior during recall but also increased exploration time compared to the first CD1 exposure (Fig. 8f). These results suggest a model in which 5-HT release in the OT during the recall exposure facilitates social avoidance behavior while its inhibition promotes approach.

Based on this, we hypothesized that 5-HT inhibition might lead to the disinhibition of DA release within the OT, promoting approach behavior. To directly test this, we combined a retrograde virus expressing Jaws (sAAV-retro/2-hSyn1-Jaws_KGC_EGFP_ERES-WPRE-hGGp(A)) with the DA sensor (pAAV-hsyn-dLight1.2) expression in the OT. Additionally, we implanted optic fibers in both the DR and OT (Fig. 8i, Supplementary Fig. 7c). This setup allowed us to simultaneously inhibit DR-OT neurons in a closed-loop manner, as previously described, and monitor DA release within the OT during the behavioral task. Intriguingly, we observed that under this inhibition, approach behavior to the enclosure containing the CD1 mouse during the recall exposure was accompanied by DA release in the OT (Fig. 8j–n), while during habituation and first exposure to CD1, no difference was found (Supplementary Fig. 7d, f).

Finally, when we specifically stimulated VTA to OT pathway with optogenetic stimulation during the recall phase, we found that there is no avoidance compared to the first exposure (Supplementary Fig 7d–g). Together, these findings reveal a dynamic interplay between 5-HT and DA in the olfactory tubercle, where 5-HT release promotes social avoidance behavior, while its inhibition unmasks DA signaling that drives social approach, suggesting a crucial neuromodulatory balance in regulating adaptive social responses.

## Discussion

Our study reveals a critical role for the OT in mediating social threat assessment and the expression of social threat-related approach and avoidance behaviors in mice. Using an odor-based paradigm, we demonstrated that mice initially encountering a restrained conspecific that subsequently becomes aggressive exhibit robust social avoidance upon re-exposure. This behavioral adaptation depends on OT activity during recall and involves distinct neuromodulatory and synaptic mechanisms. Specifically, we uncovered a dynamic interplay between serotonin and dopamine within the OT and identified recall-dependent synaptic plasticity at BLA-OT synapses, suggesting that social threat memory formation engages long-lasting circuit modifications.

Our findings position the OT as a central hub integrating olfactory information with motivational and emotional states to facilitate adaptive social behaviors. While the amygdala and hippocampus have traditionally been the focus of threat response studies^9,10,20,21^, our work extends this circuit to include the OT, a component of the ventral striatum that plays a role in odor-guided motivated behaviors. The OT shares significant anatomical and functional similarities with the Nucleus Accumbens: It is primarily composed of medium spiny neurons expressing either *Drd1* or *Drd2* dopamine receptors^22^, receives a wide range of inputs from cortical and amygdalar areas, including the BLA^23^, and it is densely innervated by both dopaminergic inputs from the VTA and serotoninergic inputs from DR^24^. Given the established role of OT in olfactory experience-dependent acquisition of behavior^15,25,26^ and our behavioral task’s reliance on odor cues, we focused our investigation on this region of the VS. While we did not segregate signals from D1- and D2-expressing MSNs, the low variability in our recordings and the absence of phasic dopamine release during recall suggest that both populations contribute similarly to the observed behavior. Critically, chemogenetic inhibition of OT activity during recall prevented avoidance behavior, likely by interfering with memory retrieval.

Remarkably, our findings indicate that OT inhibition during recall prevented avoidance behavior, whereas effects on freezing were more modest and did not reach statistical significance, suggesting that these two defensive responses may at least partially dissociate and could be regulated by distinct neural mechanisms. However, because we did not directly quantify the spatial extent and degree of OT inhibition, our data do not exclude a contribution of OT to freezing responses, and future work with more temporally precise and spatially restricted manipulations will be needed to fully define this role. Freezing is often considered an automatic, reflexive response to immediate threats, associated with “fear” circuits in the brain, especially the amygdala and periaqueductal gray^27,28^. In contrast, avoidance is a more active, strategic behavior that may involve higher-order processing, particularly in situations where the threat is not immediate but recalled from memory. This distinction is supported by studies showing that the amygdala is crucial for both freezing and avoidance but that other regions, like the prefrontal cortex and striatum, modulate more flexible, context-dependent responses such as avoidance^29^. The OT, as part of the ventral striatum, may play a specific role in modulating this type of context-dependent, learned avoidance behavior rather than freezing. By linking sensory cues (such as olfactory signals from a previously aggressive conspecific) to motivational states, the OT may facilitate adaptive decisions on whether to approach or avoid the conspecific based on prior experience. In our study, the persistence of freezing behavior despite OT inhibition suggests that the OT is not essential for this threat response but is crucial for encoding and expressing learned social avoidance.

Recent work has begun to clarify the organization of BLA outputs to ventral striatal subregions, including the tubular striatum/olfactory tubercle (TuS/OT) and the nucleus accumbens (NAc). Sniffen & Ryu and colleagues identify genetically defined BLA neuron populations (linked to dopamine receptor–related transcriptional programs) that form parallel pathways to distinct ventral striatal targets and bidirectionally modulate negative emotional states and learned fear-related behaviors depending on pathway identity^30^. In this context, we tested whether the effect reflects a broader BLA→ventral striatum influence: NAc inhibition during recall did not prevent avoidance, supporting a preferential requirement for OT in our paradigm. Given that BLA neurons can collateralize across ventral striatal targets, future intersectional strategies isolating OT-dedicated BLA → OT projections will be needed to resolve how signals are distributed across TuS/OT and NAc during threat recall.

Building upon this anatomical framework, we investigated the specific circuit mechanisms underlying the role of OT in threat processing. The OT receives diverse inputs, including sensory projections from the olfactory bulb, as well as intracortical associative inputs from the olfactory cortex and amygdala^23^. Previous studies have demonstrated that both sensory and intracortical inputs to the OT exhibit learning-dependent plasticity, suggesting that synaptic modifications underlie the selective activation of OT domains and subsequent acquisition of appropriate motivated behaviors^31^. Our investigation revealed that excitatory inputs from the BLA to OT were specifically activated during recall and underwent experience-dependent synaptic plasticity. This plasticity emerged rapidly during the recall phase and persisted for at least 24 hours after extinction training, suggesting a stable modification of the circuit. Intriguingly, we observed that the BLA-OT pathway was also activated during grooming behavior, suggesting it may convey a broader salience- or attention-like signal for behaviorally relevant social events, which is then recruited during recall to bias OT processing toward avoidance. This observation aligns with previous findings showing that ventral striatal islands of Calleja neurons control grooming in mice^32^. These results raise the possibility that the BLA to OT pathway may serve a broader role in regulating social threat-related approach and avoidance strategies, as well as grooming, rather than signaling threat memory in isolation. The temporal overlap between threat assessment and grooming-related activation suggests a complex integration of different behavioral states, though future studies will be needed to test this hypothesis directly.

We next investigate the role of neuromodulatory systems in adapting behavior during recall. The dynamic interaction between serotonin and dopamine within the OT reveals a sophisticated mechanism for behavioral regulation. During threat assessment, we observed that serotonin release facilitates avoidance behavior, while its inhibition leads to dopamine release and promotes approach behavior. This functional opponency aligns with existing studies on neuromodulatory influences in decision-making^33^ and extends beyond simple behavioral switching, suggesting a complex integration of past experiences with current contextual assessment. The temporal dynamics of this neuromodulatory balance are particularly noteworthy - the absence of baseline dopamine signaling, followed by its emergence only under serotonin suppression, indicates a hierarchical organization that may serve to prevent inappropriate approach behavior during periods of uncertainty or potential threat.

At a physiological level, our data are consistent with a model in which DR-derived 5-HT constrains DA-dependent approach signals in the OT through a combination of pre- and postsynaptic mechanisms^33^. Serotonin receptors expressed on OT medium spiny neurons are well positioned to modulate their excitability and integration of dopaminergic input, for example by biasing the relative recruitment of D1- and D2-type populations and thereby shifting the balance between approach and avoidance states^34,35^. In addition, 5-HT receptors on presynaptic terminals of DA afferents, or on local interneurons, could directly regulate DA release probability and/or the gain of DA-sensitive synapses^36,37^. In this framework, strong DR → OT activation during social threat recall would both enhance the responsiveness of avoidance-promoting OT ensembles and suppress DA-driven approach signals, whereas inhibition of DR → OT input would release this brake, allowing DA transients to emerge during approach. Although our experiments do not identify the specific receptor subtypes or cell classes involved, neither take in consideration the possibility of corelease^38^, they provide a circuit-level constraint that future work can use to dissect the precise synaptic and molecular interactions between 5-HT and DA in the OT.

While both neuromodulatory mechanisms and synaptic plasticity occur within the OT, our findings suggest they operate independently and serve distinct functions in threat processing. The serotonin-dopamine balance appears to acutely control behavioral responses during recall, with serotonin promoting avoidance and dopamine facilitating approach behavior. In contrast, the BLA-OT synaptic plasticity emerges specifically after the recall phase, probably independently of these neuromodulatory effects. This temporal and functional separation suggests a two-step process: First, rapid neuromodulatory control of immediate behavioral responses, followed by recall-dependent synaptic modifications that may encode memory retrieval itself. The specific trigger for this recall-induced plasticity likely involves mechanisms beyond serotonin and dopamine signaling, raising intriguing questions about the molecular pathways involved. Whether similar temporally structured arrangements exist for other types of social memories within the OT remains an open question that could provide insights into how the brain separates acute behavioral control from longer-term memory modifications.

The stimulus-specific synaptic plasticity observed at BLA-OT synapses following recall exposure suggest the role of OT in the recall and expression of social threat memories rather than initial threat encoding. This specificity ensures precise behavioral adaptation to relevant social cues, with plasticity persisting even after extinction. These findings align with the theory of memory reconsolidation, which proposes that retrieved memories become temporarily labile and susceptible to modification based on new experiences^39,40^. The persistence of synaptic changes after extinction is particularly noteworthy, as it suggests that information about non-threatening encounters may be incorporated into the same circuit without erasing the original threat trace^41^. The temporal dynamics of these synaptic modifications raise important questions about the molecular mechanisms involved. How does the maintenance of these synaptic modifications contribute to the formation of new safety memories while preserving information about potential threats? Understanding these mechanisms could provide crucial insights into pathological conditions where threat memories persist inappropriately.

While our paradigm provides valuable insights into social threat processing, several limitations should be considered. First, our use of a confined space may influence the behavioral strategies available to the mice. Future studies using open-field environments could help determine whether the OT’s role generalizes across different contexts. Second, while our ethogram captures core defensive strategies, the integration of high-resolution, high-dimensional behavioral mapping could further refine our understanding of social threat dynamics. Traditional manual scoring often collapses complex, fluid actions into discrete, broad categories, potentially obscuring the fine-grained ‘behavioral syllables’ that constitute social interaction. Modern computational approaches—such as unsupervised clustering of pose data (e.g., MoSeq^42^) derived from deep-learning-based tracking—offer an objective dissection of these responses by identifying stereotyped motifs and their transition probabilities. Such resolution would allow us to determine whether neurobiological modulations, such as those involving the OT system, alter the fundamental grammar and structure of defensive behavior or merely shift the threshold for its expression. Finally, our focus on male mice leaves open the question of potential sex differences in these mechanisms, particularly given known sexual dimorphisms in social behavior and stress responses.

Our findings also raise important questions about how these mechanisms compare to those involved in other forms of social threat processing. Unlike traditional social defeat paradigms that involve chronic stress, our paradigm reveals acute mechanisms of threat assessment and changes in the expression of social threat memories. However, it remains unclear how these acute mechanisms might interface with or transform under conditions of chronic social stress. Moreover, while our findings demonstrate the importance of olfactory cues, future studies should investigate how the OT integrates multiple sensory modalities during threat assessment. Additionally, several mechanistic questions remain unresolved. What is the precise temporal relationship between neuromodulator release and synaptic plasticity? How do these mechanisms operate in more complex social scenarios involving multiple conspecifics or competing motivational states? How long do these synaptic modifications persist, and what determines their stability?

Our findings have significant implications for understanding and treating psychiatric conditions characterized by maladaptive threat processing. The identified neuromodulatory balance between serotonin and dopamine suggests potential therapeutic strategies for conditions like anxiety disorders and post-traumatic stress disorder. The persistence of synaptic modifications after extinction raises possibilities for enhancing exposure therapy outcomes through neuromodulatory interventions. Understanding how these mechanisms might be altered in psychiatric conditions could lead to more targeted therapeutic approaches.

In conclusion, by identifying the OT as a critical neural substrate for social threat assessment and memory reconsolidation, and uncovering the underlying neuromodulatory and synaptic mechanisms, our study advances the understanding of adaptive social behavior. The dynamic interplay between serotonin and dopamine, together with pathway-specific synaptic plasticity, provides a mechanistic framework for understanding how animals navigate complex social environments. The persistence of these modifications after extinction suggests a sophisticated system for maintaining threat-related information while allowing for behavioral flexibility. These insights not only enhance our basic understanding of social cognition but also provide a foundation for developing targeted interventions in disorders characterized by maladaptive threat processing

## Methods

### Animals

The study was conducted with adult (8–16 weeks old) male WT and transgenic mice in C57BL/6 J background. For GAD+ neurons-specific manipulation Gad2-IRES-Cre (Gad2tm2(creZjh)/J) mice from Charles River were used. For serotoninergic neuron specific manipulation SERT-cre (B6.129(Cg)-Slc6a4tml(cre)Xz/J) mice from Charles River were used. For dopaminergic neuron specific manipulation DAT-IRES-cre (B6.SJL-Slc6a3tm1.1(cre)Bkmn/J) mice from Charles River were used. Concerning expression-mediated staining CreERt2 (Fos<tm2.1(icre/ERT2)Luo > ) mice were used (in-house breed). An additional line referred to as CD1 (Crl:CD1(ICR)) was used for stimuli involved in aggressive behaviors, they were obtained from Charles Rivers. CD1 stimuli correspond to retired breeders more than 4 months old and they were screened for aggressiveness according to Golden et al. 2011. All the animals were grouped housed (2–5 per cage) and stayed with the same cage mates since they were weaned (P21–P28). They stayed under a 12 h light-dark cycle (7:00 a.m.–7:00 p.m.). All physiology and behavior experiments were performed during the light cycle. All the procedures performed at UNIGE complied with the Swiss National Institutional Guidelines on Animal Experimentation and were approved by the Swiss Cantonal Veterinary Office Committees for Animal Experimentation.

### Surgeries

For all the surgical procedures described hereafter, mice were anesthetized with a mixture of oxygen (1 L/min) and isoflurane 2% (Baxter AG, Vienna, Austria) and placed in a stereotactic frame (Angle One; Leica, Germany). The skin was locally anesthetized with 40–50 μL lidocaine 0.5% and disinfected with betadine.

#### Fos CreERt2 mice

Injections of AAVrg-Flex-tdTomato (retrograde virus, 28306-AAVrg, Addgene) were performed in male CreERt2 mice at 17 weeks of age. Unilateral craniotomy (1 mm in diameter) was then performed over the Olfactory Tubercle (OT) at the following stereotactic coordinates: AP + 1.2 mm, ML ± 1.1 mm, DV − 5.3 mm from Bregma. The virus was injected via a glass micropipette (Drummond Scientific Company, Broomall, PA) into the OT for a total volume of 200 nL. The virus was incubated for 4 weeks before performing the behavioral tasks.

#### Bicolor

Injections of AAVrg-ef1a-DO-DIO-TdTomato-eGFP (Addgene plasmid 37120; http://n2t.net/addgene:37120; RRID:Addgene_37120) were performed on GAD-Cre male and female mice at 10–12 weeks. Unilateral craniotomy was performed over OT (AP + 1.2 mm, ML ± 1.1 mm, DV − 5.3 mm from Bregma). The volume of virus injected was 200 nL. The virus was incubated for 3-4 weeks before performing the perfusion and acquisition of the images.

#### SERT-cre mice

Injections of ssAAV-9/2-hSyn1-dlox-tdTomato(rev)-dlox-WPRE-bGHp(A) (v284-9, Viral vector ETH Zurich) were performed on SERT-Cre male mice at 10–12 weeks. Unilateral craniotomy was performed over DR (AP − 4.6 mm, ML 0 mm, DV − 2.8 mm from Bregma). The volume of virus injected was 200 nL. The virus was incubated for 3-4 weeks before performing the perfusion and acquisition of the images.

#### Chemogenetics

Injections of ssAAV-5/2-hSyn1-hM4D(Gi)-mCherry (v107-5, Viral vector ETH Zurich) or ssAAV-5/2-hSyn1-mCherry (v253-5, Viral vector ETH Zurich) were performed on WT male mice at 10–12 weeks. Bilateral craniotomy was performed over OT (AP + 1.2 mm, ML ± 1.1 mm, DV − 5.3 mm from Bregma) or Nucleus accumbens (AP + 1.2 mm, ML ± 1.1 mm, DV − 4.2 mm from Bregma). The volume of virus injected was 200 nL. The virus was incubated for 3–4 weeks before performing behavioral experiments.

#### Patch clamp

Injections were performed on WT male mice at 10–12 weeks. Bilateral craniotomy was performed over the Basolateral amygdala (AP –1.6 mm, ML ± 3.1 mm, DV − 4.1 mm from Bregma) injecting rAAV5-CamKII-hChr2-eYFP-WPRE (26969-AAVrg, Addgene). The volume of the virus injected was 300 nL. The virus was incubated for 3-4 weeks before performing the experiments.

#### Fiber photometry experiments

Injections were performed on WT male mice at 10–12 weeks. A unilateral craniotomy was performed over the OT injecting pAAV-hsyn-dLight1.2 (111068, Addgene), ssAAV5/2-hsyn1-GCamp7s-WPRE-SV40 (v406-5, Viral vector ETH Zurich), ssAAV-retro/2-mCamKIIa-jGCamp7s-WPRE-bGHp (v482-retro, Viral vector ETH Zurich), or pAAV-hsyn1-5HT3.0 (PT-4724, Brain VTA (Wuhan) Co LTD). The volume of the virus injected was 300 nL. Following the injection of viruses an optic fiber (∅ 200 μm) was implanted and cemented with C&B-Metabond® and dental acrylic cement. A second unilateral craniotomy, for mice injected with ssAAV-retro/2-mCamKIIa-jGCamp7s-WPRE-bGHp, was performed over the Basolateral amygdala (AP –1.6 mm, ML ± 3.1 mm, DV − 4.1 mm from Bregma) where an optic fiber (∅ 200 μm) was implanted and cemented with C&B-Metabond® and dental acrylic cement. The virus was incubated for 3–4 weeks before performing the experiments.

#### Optogenetic experiments

Injections were performed on WT male mice at 10–12 weeks. Craniotomy was performed over the OT injecting sAAV-retro/2-hsyn1-Jaws_KGC_EGFP_ERES-WPRE-hGGp(A) (v387-retro, Viral vector ETH Zurich) or retro pAAV-hsyn-EGFP (50465-AAVrg, Addgene). The volume of the virus injected was 300 nL. A second craniotomy was performed over the Raphe Nuclei (AP −4.6 mm, ML 0 mm, DV −2 mm) where an optic fiber (∅ 200 μm) was implanted and cemented with C&B-Metabond® and dental acrylic cement. The virus was incubated for 3–4 weeks before performing the experiments.

Injections of ssAAV-retro/2-hEF1a-dlox-hChR2(H134R)_EYFP(rev)-dlox-WPRE-hGHp(A) (v214-retro, Viral vector ETH Zurich) were performed on DAT IRES-Cre male mice at 10–12 weeks. Unilateral craniotomy was performed over OT (AP + 1.2 mm, ML ± 1.1 mm, DV − 5.3 mm from Bregma). The volume of virus injected was 200 nL. A second craniotomy was performed over the Ventral Tegmental Area (AP −3.2 mm, ML 0.9 mm, DV −4.2 mm) where an optic fiber (∅ 200 μm) was implanted and cemented with C&B-Metabond® and dental acrylic cement.The virus was incubated for 3–4 weeks before performing the perfusion and acquisition of the images.

### Anatomy

#### Anatomical tracing of active inputs using CreRT2 animals

After the social avoidance corridor task, we wait 4 weeks for the expression of the virus. Then, the animals were sacrificed by lethal injection of pentobarbital and transcardially perfused with phosphate buffer solution (PBS) 1x followed by 4% paraformaldehyde prepared in PBS 1x. The brain was then extracted and stored at 4 °C overnight to post fixate in the same solution. The relevant regions of the brain were sliced in 50 μm thick coronal slices (Leica VT1000S). Then the slices were mounted and covered with mounting medium with DAPI and cover slides.

#### CreERt2 mice cell counting

Following a qualitative analysis of the viral expression, only a subset of regions was taken into consideration for quantitative cell counting; these regions being the lateral olfactory tract (LOT), basolateral amygdala (BLA), piriform cortex and the anterior insular cortex (AIC). Image processing and cell counting were performed using Zen 3 (Blue version) software using built-in cell counting and regions of interest (ROI) measurement features. Structural boundaries of the abovementioned regions were identified with anatomical landmarks according to a brain atlas (Franklin & Paxinos, 2008). For each region, positive cells and area were measured. The density index was then calculated in Excel by dividing the positive cells of each structure by its area (in square mm). Every region was counted a similar number of times on different slices by two independent experimenters. Individual data were then pulled together and analyzed using GraphPad Prism 7.

#### Representative photos of CreERt2 mice

For representative photos of Crert2 mice, we did an immunohistochemistry against td-Tomato. We incubated 48 h at 4 °C with rat anti-tdTomato (16D7, Kerafast) diluted 1:500 in a solution of 3% bovine albumin serum and 0.3% Triton X-100 prepared in PBS 1x. After primary incubation, the tissue was rinsed 3 times with PBS 1x and incubated at room temperature for 1.5 h with donkey anti-rat IgG H&L (Alexa Fluor® 555) diluted 1:500 in a solution of 0.25% Tween- 20 prepared in PBS 1x.

#### Bicolor cell counting

Images were acquired with an LSM700 confocal microscope, LSM 800 Airyscan confocal microscope or Axioscan-Z1 epifluorescence slide scanner. Only images captured with the Axioscan-Z1 without immunohistochemistry were used for cell counting. Image processing and cell counting were performed using Zen 3 (Blue version) software using built-in cell counting and regions of interest (ROI) measurement features. Structural boundaries of the abovementioned regions were identified with anatomical landmarks according to a brain atlas (Franklin & Paxinos, 2008). For each region positive cells and area were measured. Density index was then calculated in Excel by dividing the positive cells of each structure by its area (in square mm). Every region was counted a similar number of times on different slices by two independent experimenters. Individual data were then pulled together and analyzed using GraphPad Prism 7.

#### Bicolor representative pictures

For representative photos of the bicolor virus approach, brains infected with AAVrg-ef1a-DO-DIO-TdTomato-eGFP were incubated overnight at 4 °C with rabbit anti-GFP (A-11122, Thermo Fisher Scientific) and rat anti-tdTomato (16D7, Kerafast) diluted 1:500 in a solution of 3% bovine albumin serum and 0.3% Triton X-100 prepared in PBS 1x. After primary incubation, the tissue was rinsed 3 times with PBS 1x and incubated at room temperature for 1.5 h with goat anti-rabbit IgG H&L (Alexa Fluor® 488) and donkey anti-rat IgG H&L (Alexa Fluor® 555) diluted 1:500 in a solution of 0.25% Tween-20 prepared in PBS 1x. After staining procedures or simple slicing (injection or implantation site post-hoc confirmation), slices were rinsed 3 times in PBS 1x, mounted on glass slides, and covered with a mounting medium with DAPI and cover slides.

### Behavior

#### Social threat recognition test

The arena of the social threat avoidance test consists of a corridor arena (60 cm × 15 cm x 30 cm) wherein on one of the sides we positioned an opaque dark plastic enclosure with small openings (diameter 0.3 cm) which allowed an exchange of only auditory and olfactory but not visual and tactile cues between mice. This enclosure was closed at the base and the top. To prevent mice from going past the cylinder, a PVC sheet was cut to the dimensions of the cylinder and the arena which was then placed at the level of the cylinder to create a separation. After a 10 min habituation period with the empty enclosure, a pre-screened aggressive CD1 mouse was introduced into it for 1^st^ exposure to CD1. Subsequently, the experimental mouse and the CD1 were transferred to the home cage of the CD1 for 10 min for the aggression phase. Mice were then divided by a custom-made plexiglass wall that allowed an exchange of sensory information but not direct physical contact. Mice were then returned to their home cage for 1 h. In the final recall phase, the mice were reintroduced into the arena, facing the same CD1 mouse as in the initial exposure. Every session was video-tracked and recorded using Ethovision XT (Noldus, Wageningen, the Netherlands), which provided an automated recording of the entries around the enclosure, the time around the enclosure, the distance moved, and the velocity. The mice were considered to explore the enclosure when their nose was directed towards the enclosure at a distance of less than approximately 2 cm. Several types of behaviors were then manually scored using built-in features within Ethovision XT. The time spent actively investigating the enclosure was measured. Freezing was defined as complete immobility (the absence of all movement except for respiratory-related activity) lasting ≥ 2 s^43^. This 2 s threshold is a standard metric used to exclude brief, non-defensive pauses in locomotion while reliably capturing sustained defensive immobility. The time spent rearing which corresponds to mice briefly standing on their hind legs^44^ was quantified as a measure of exploratory behaviors. Finally, grooming behaviors were also manually scored.

#### Chemogenetic inhibition

Following the viral injection procedures previously described, mice were subjected to the social threat avoidance test. To ensure peak ligand bioavailability coincided with the behavioral phase of interest, mice were administered CNO (3 mg/kg, i.p.) at a lead time calculated to reach maximum concentration during the target experimental window (see Figures).

Mice tested for the innate fear paradigm, underwent to a behavioral paradigm consisting of a 10 min habituation in the corridor arena followed by a 10 min exposure to a threat (TMT)

#### Fos CreERt2 mice

Before the social threat avoidance test and after the viral injections, CreERt2 mice were habituated 10 minutes for 5 consecutive days in the corridor and a new home cage (35 cm × 19 cm x 13 cm) prior to the behavioral experiments, each “habituation cage” was specific for one animal during all sessions. At the end of each habituation session, they were injected intraperitoneally with a saline solution (1 mL/Kg) according to the protocol described by Ye et al. 2016. The day of the test, after an initial period of 15 min of habituation, they performed the social threat recognition test. After that, they received an intraperitoneal injection of 4-OH Tamoxifen (10 mg/Kg) to induce Cre recombinase expression. They were then left in their “habituation cage” for 4 h to ensure the level of cre expression was specific to the social interaction time window (Ye et al. 2016). After this period, they were put back in their home cages.

#### Closed-loop optogenetic inhibition

Following the procedures for viral injection and optic fiber implantation previously described, mice were subjected to the social threat avoidance test. During the recall phase, light was administered through a real-time closed-loop system triggered by the animal’s spatial coordinates. A virtual circular “trigger zone” was defined with a diameter 5 cm larger than the enclosure (øzone = øenclosure +5 cm). This 5 cm threshold was specifically chosen for the online trigger to ensure high reliability; while social interaction is offline-analyzed at a 2 cm proximity, the wider 5 cm online buffer accounts for potential tracking jitter or “swaps” between the animal’s nose and tail points. This ensures that the laser remains active whenever any part of the animal’s head or body center is in close proximity to the social stimulus. The presence of the mouse within this trigger zone, identified by the tracking software (Ethovision XT), initiated a signal conversion into a TTL pulse, which in turn triggered the delivery of laser light. For Jaws-expressing mice and their respective eYFP-controls, we applied continuous 640 nm light (BioRay Laser, Coherent). For ChR2-expressing mice and controls, the TTL pulse triggered a high-frequency stimulation protocol consisting of 30-pulse bursts at 20 Hz (5 ms pulse width) using a 488 nm laser (BioRay Laser, Coherent). All hardware was integrated via Imetronic (Pessac, France) control modules.

### Ex-vivo patch clamp recordings

We prepared 250 μm thick coronal slices containing OT. Slices were kept in artificial cerebrospinal fluid containing 119 mM NaCl, 2.5 mM KCl, 1.3 mM MgCl_2_, 2.5 mM CaCl_2_, 1.0 mM NaH_2_PO_4_, 26.2 mM NaHCO_3_, and 11 mM glucose, bubbled with 95% O2 and 5% CO_2_. Slices were maintained for 30 min in a bath at 30 °C and then at room temperature. Whole-cell voltage-clamp recording techniques were used (37 °C, 2–3 ml/min, submerged slices) to measure the holding currents and synaptic responses. The internal solution contained 130 mM CsCl, 4 mM NaCl, 2 mM MgCl_2_, 1.1 mM EGTA, 5 mM HEPES, 2 mM Na2ATP, 5 mM sodium creatine phosphate, 0.6 mM Na3GTP, and 0.1 mM spermine. Access resistance was monitored by a hyperpolarizing step of −4 mV at each sweep, every 10 s. The cells were recorded at the access resistance from 5 to 20 MΩ for MSN. Data were excluded when the resistance changed more than 25% across the recording. Synaptic currents were evoked by optogenetic stimulation at 0.1 Hz and 1–3 ms of duration. The experiments were conducted in the presence of GABAA receptor antagonist picrotoxin (100 mM); The AMPA/NMDA ratio was calculated by dividing the synaptic response at +40 mV, from the response at −60 mV. The rectification index (RI) of AMPARs is the ratio of the chord conductance calculated at negative potential (–60 mV) divided by the chord conductance at positive potential ( + 40 mV) after the AMPA current isolation by D-APV (50 μM) bath application. Representative example traces are shown as the average of 15–20 consecutive EPSCs typically obtained at each potential. The rectification index of AMPARs is the ratio of the chord conductance calculated at negative potential (−60 mV) divided by the chord conductance at positive potential ( + 40 mV). The Synaptic responses were collected with a Multiclamp 700B-amplifier (Axon Instruments), filtered at 2.2 kHz, digitized at 10 Hz, and analyzed using Igor Pro 6.3 and 8.0 software (Wavemetrics).

### Analysis

#### Analysis of Behavioral transitions

Behavioral sequences were analyzed using a custom Python pipeline. We calculated first-order transition probability matrices for each subject by determining the frequency of transitions between consecutive bouts (e.g., Behavior A → B) relative to the total number of transitions from the initial state. This included both cross-behavioral and self-transitions. Group-level results were generated by aggregating individual matrices to compute an averaged transition probability matrix, which was visualized via heatmaps to identify shifts in behavioral flow across experimental phases.

#### Analyses of Fiber photometry data

Fiber photometry data were acquired and processed using Doric Studio software (Doric Lenses, Quebec, Canada). Fluorescence was recorded at 12 kHz using dual-channel excitation: a signal channel (470 nm) for calcium-dependent activity and an isosbestic channel (405 nm) to monitor calcium-independent artifacts, such as photobleaching and motion. To calculate the relative change in fluorescence (ΔF/F), the 405 nm isosbestic signal was used as a dynamic baseline. Within Doric Studio, the 405 nm signal was fitted to the 470 nm signal using a least-squares linear regression. This fitted trace (F0) was used to calculate the ratiometric ΔF/F as (F470 − F0)/F0. The resulting traces were exported for analysis using custom Igor Pro (Wavemetrics, Lake Oswego, OR, USA) scripts. Peri-Event Time Histograms (PETHs) were constructed by time-locking the ΔF/F signal to behavioral events identified by Ethovision XT (Noldus, Wageningen, the Netherlands). Social investigation was defined as entry into a virtual circular zone centered on the enclosure (øzone=øenclosure+5 cm). Non-social behaviors, including rearing, grooming, and freezing, were similarly used to evaluate circuit activity across diverse behavioral states.

#### Statistical analysis

Statistical analysis was conducted with GraphPad Prism 7 and 8 (San Diego, CA, USA). Statistical outliers were identified with the ROUT method (Q = 1) and excluded from the analysis. The normality of sample distributions was assessed with the Shapiro–Wilk criterion and when violated non-parametric tests were used. When normally distributed, the data were analyzed with independent t-tests, one-sample t-tests, one-way ANOVA, and repeated measures (RM) ANOVA as appropriate. When normality was violated, the data were analyzed with Mann–Whitney or Wilcoxon tests, while for multiple comparisons, Kruskal–Wallis or Friedman test was followed by Dunn’s test. For the analysis of variance with two factors (two-way ANOVA, RM two-way ANOVA, and RM two-way ANOVA by both factors), normality of sample distribution was assumed, and followed by Sidak or Tukey post hoc test. Data are represented as the mean ± SEM and the significance was set at 95% of confidence.

### Reporting summary

Further information on research design is available in the Nature Portfolio Reporting Summary linked to this article.

## Supplementary information

Supplementary Information

Reporting Summary

Transparent Peer Review file

## Source data

Source Data

## Data Availability

All the data are in the manuscript or in supplementary material. Source data are provided with this paper or are available in the following link: 10.5281/zenodo.19352359. Further data supporting the findings are available upon request. Source data are provided with this paper.

## Code availability

Innovative codes used in this study are available in the following link: 10.5281/zenodo.19352359.
