# Supplementary material for: Olfactory tubercle mediates adaptive social behavior by controlling threat assessment and the expression of social threat memories during recall in male mice
Source: Nat Commun. 2026 May 19;17:6597. doi: 10.1038/s41467-026-73268-w (PMC13381864; doi:10.1038/s41467-026-73268-w)
Supplement: Supplementary file 1 — Supplementary Information [file 41467_2026_73268_MOESM1_ESM.pdf]

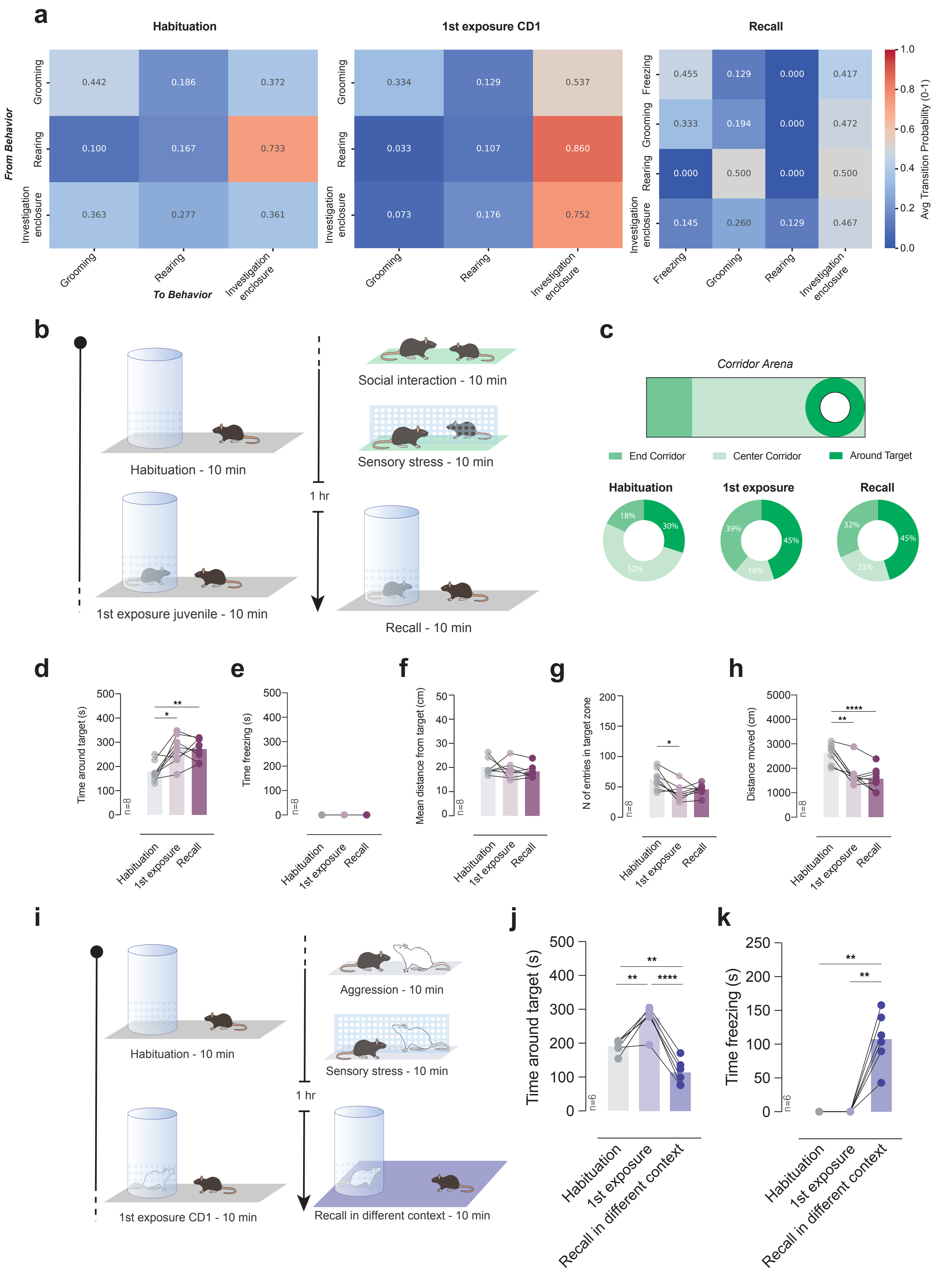

Supplementary Fig. 1, Casarotto et al

Supplementary Fig. 1. (a) Heatmaps representing the average transition probabilities between behavioral states during Habituation, 1st exposure (CD1), and recall phases. The y-axis denotes the preceding behavior (From Behavior), and the x-axis denotes the subsequent behavior (To Behavior). (b) Schematic representation of experimental test. (c) Top: Schematic representation of the experimental arena partitioned into three zones: End Corridor (medium green), Center Corridor (light green), and Around Target (dark green). Bottom: Pie chart representing the mean percentage of time spent by mice in each zone during three distinct behavioral phases. (d) Time around enclosure containing stimulus (RM one-way ANOVA: $F_{1.703, 11.92} = 13.40$, $p = 0.0012$, followed by Bonferroni multiple comparison post hoc test). (e) Time freezing during the test. (f) Mean distance between nose point of the experimental mouse and the center of the target during the test (RM one-way ANOVA: $F_{1.220, 7.321} = 0.5453$, $p = 0.5177$, followed by Bonferroni multiple comparison post hoc test). (g) Number of entries in target zone during the test (RM one-way ANOVA: $F_{1.691, 11.83} = 6.290$, $p = 0.0166$, followed by Bonferroni multiple comparison post hoc test). (h) Distance moved in apparatus (RM one-way ANOVA: $F_{1.234, 8.641} = 36.72$, $p = 0.0001$, followed by Bonferroni multiple comparison post hoc test). (i) Schematic representation of experimental test. (j) Time around enclosure containing stimulus (RM one-way ANOVA: $F_{1.728, 8.638} = 53.56$, $p < 0.0001$, followed by Bonferroni multiple comparison post hoc test). (k) Time freezing during the test (RM one-way ANOVA: $F_{1.000,5.000} = 43.01$, $p = 0.0012$, followed by Bonferroni multiple comparison post hoc test).

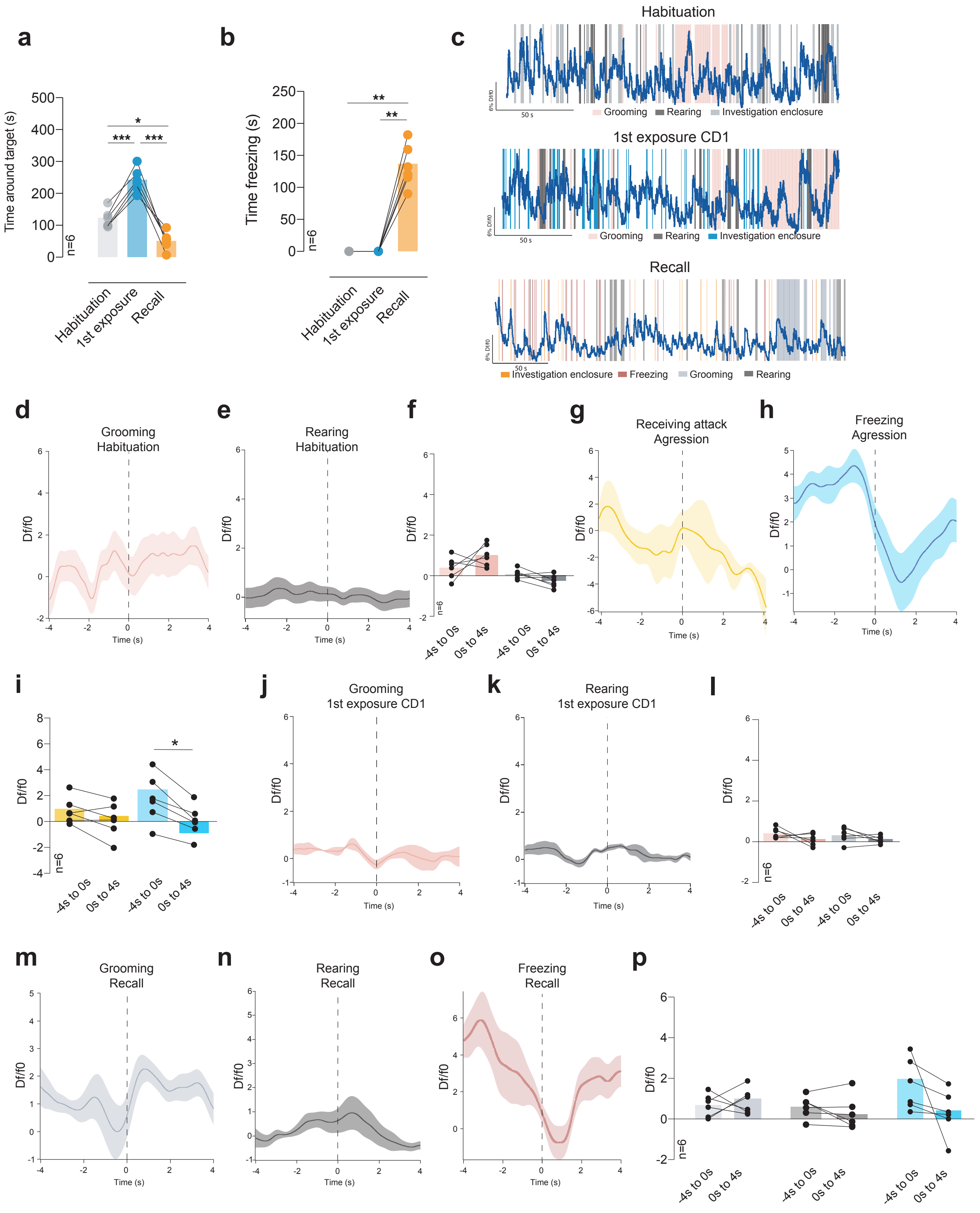

Supplementary Fig. 2, Casarotto et al

Supplementary Fig 2. (a) Time around enclosure containing stimulus (RM one-way ANOVA: $F_{1.391,\ 5.566} = 45.67$, $p = 0.0005$, followed by Bonferroni multiple comparison post hoc test). (b) Time freezing during the test (RM one-way ANOVA: $F_{1.000,\ 4.000} = 74.36$, $p = 0.0010$, followed by Bonferroni multiple comparison post hoc test). (c) Example traces of photometry signals (reported as DF/F0, see Methods) during habituation, 1st exposure and recall. Colored boxes above the traces indicate investigating the enclosure, freezing, grooming and rearing bouts. (d) Mean DF/F0 signal ±4s around grooming initiation (indicated by dashed line, 0s) during habituation. (e) Mean DF/F0 signal ±4s around rearing initiation (indicated by dashed line, 0s) during habituation. (f) Quantification of DF/F0 difference before and after grooming (pink) and rearing (gray) start for the different phases of the test (Left: paired t-test, $t1.466 = 5$, $p = 0.2026$. Right: paired t-test $t2.396 = 5$, $p = 0.0619$). (g) Mean DF/F0 signal ±4s around receiving attack initiation (indicated by dashed line, 0s) during the aggression phase of the test. (h) Mean DF/F0 signal ±4s around freezing initiation (indicated by dashed line, 0s) during the aggression phase of the test. (i) Quantification of DF/F0 difference before and after receiving attack (yellow) and freezing (blue) start for the different phases of the test (Left: paired t-test, $t1.777 = 5$, $p = 0.1357$. Right: paired t-test $t3.201 = 5$, $p = 0.0240$). (j) Mean DF/F0 signal ±4s around grooming initiation (indicated by dashed line, 0s) during 1st exposure to CD1. (k) Mean DF/F0 signal ±4s around rearing initiation (indicated by dashed line, 0s) during 1st exposure to CD1. (l) Quantification of DF/F0 difference before and after grooming (pink) and rearing (gray) start for the different phases of the test (Left: paired t-test, $t1.915 = 5$, $p = 0.1137$. Right: paired t-test $t1.382 = 5$, $p = 0.2255$). (m) Mean DF/F0 signal ±4s around grooming initiation (indicated by dashed line, 0s) during recall. (n) Mean DF/F0 signal ±4s around rearing initiation (indicated by dashed line, 0s) during recall. (o) Mean DF/F0 signal ±4s around freezing initiation (indicated by dashed line, 0s) during recall. (p) Quantification of DF/F0 difference before and after grooming (light gray), rearing (dark gray) and freezing (blue) start for the different phases of the test (Left: paired t-test, $t_5 = 1.777$, $p = 0.1357$. Middle: paired t-test, $t1.196 = 5$, $p = 0.2855$. Right: paired t-test $t_5 = 1.822$, $p = 0.1281$).

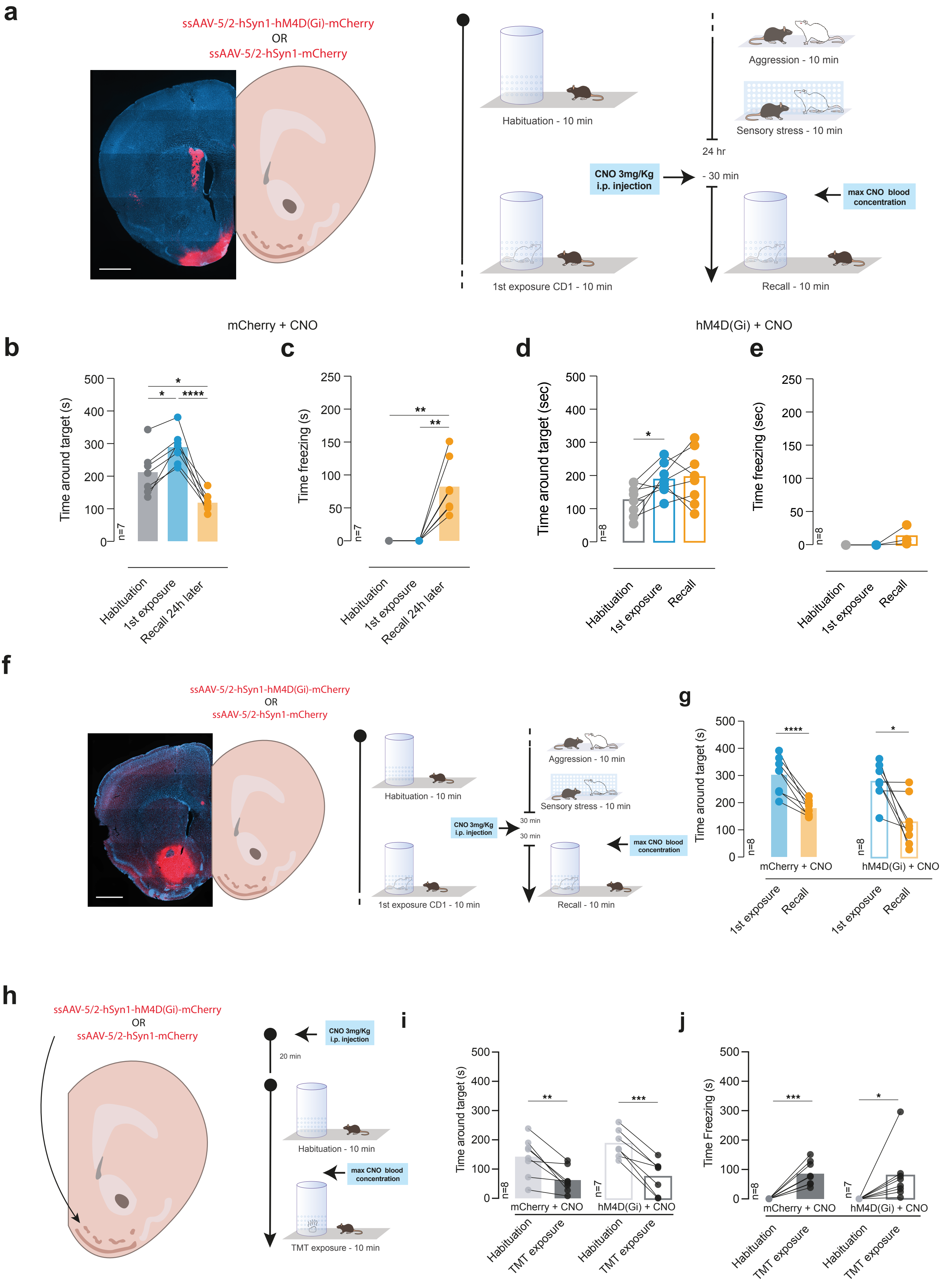

Supplementary Fig. 3, Casarotto et al, 2024

Supplementary Figure 3. (a) Left: Representative picture and schematic of the viral injection and representative image of injection site (scale bar: 750 µm). Right: Schematic of the experimental timeline. (b) Time around enclosure containing stimulus (RM one-way ANOVA: $F_{1.540,9.243} = 29.04$, $p = 0.0002$, followed by Bonferroni multiple comparison post hoc test). (c) Time freezing during the test (RM one-way ANOVA: $F_{1.000, 6.000} = 26.40$, $p = 0.0021$, followed by Bonferroni multiple comparison post hoc test). (d) Time around enclosure containing stimulus (RM one-way ANOVA: $F_{1.110,7.767} = 3.501$, $p = 0.0973$, followed by Bonferroni multiple comparison post hoc test). (e) Time freezing during the test (RM one-way ANOVA: $F_{1.000, 7.000} = 1.842$, $p = 0.2168$, followed by Bonferroni multiple comparison post hoc test). (f) Left: Representative picture and schematic of the viral injection and representative image of injection site (scale bar: 750 µm). Right: Schematic of the experimental timeline. (g) Time around enclosure containing stimulus (Left: paired t-test $t_7 = 5.961$, $p = 0.0006$. Right: paired t-test $t_5 = 3.586$, $p = 0.0116$.). (h) Left: Schematic of the viral injection. Right: Schematic of the experimental timeline. (i) Time around enclosure containing stimulus (Left: paired t-test $t_7 = 3.926$, $p = 0.00057$. Right: paired t-test $t_{7.449} = 7$, $p = 0.0003$). (j) Time freezing (Left: paired t-test $t_7 = 5.938$, $p = 0.0006$. Right: paired t-test $t_{2.456} = 7$, $p = 0.0437$).

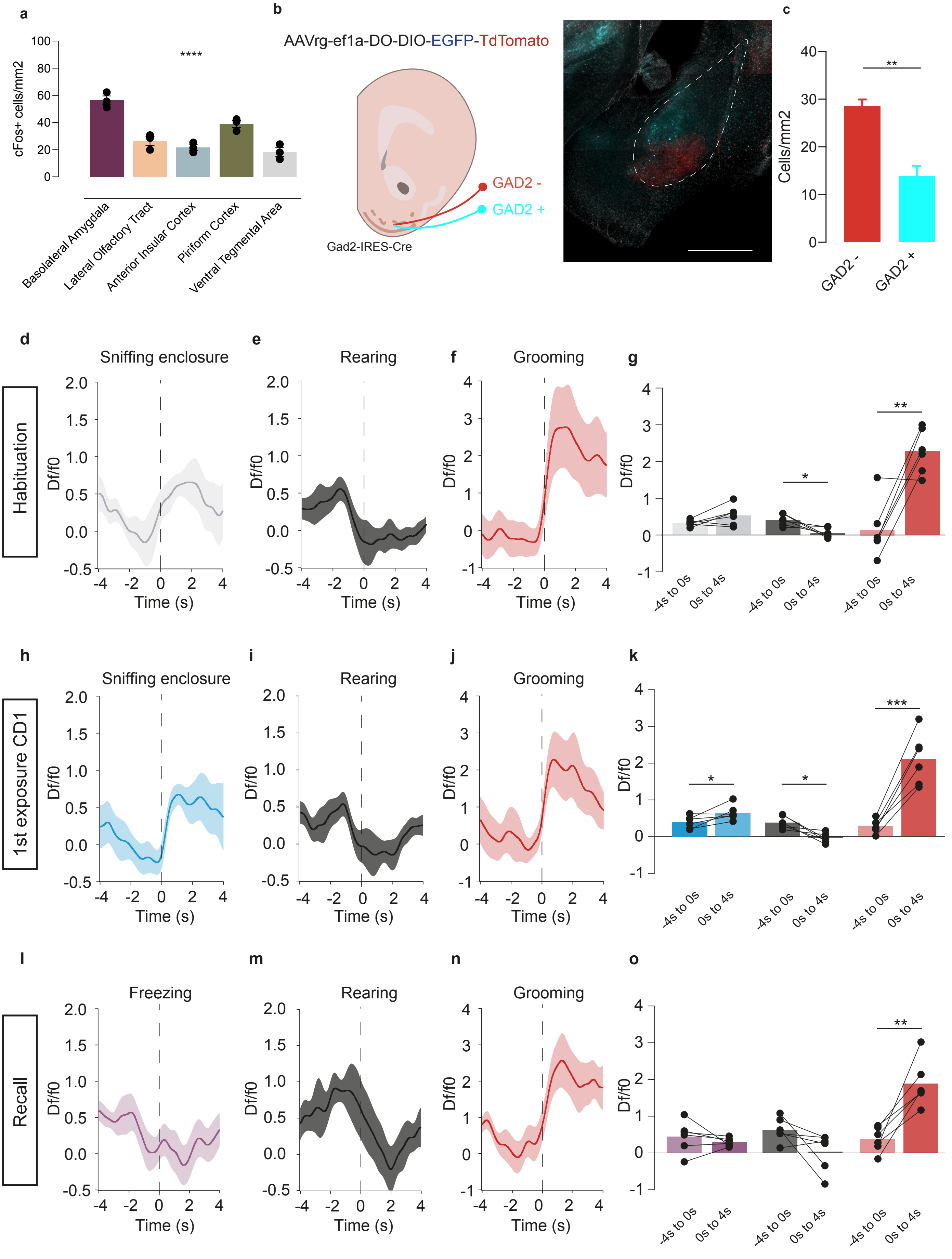

Supplementary Fig. 4, Casarotto et al, 2024

Supplementary Figure 4. (a) Number of cFos+ cells/mm2 quantified (Ordinary one-way ANOVA: $F(4, 10) = 28.45$. $p < 0.0001$). (b) Left: Schematic of the viral injection. Right: Representative pictures of inputs from basolateral amygdala. (c) Quantification of GAD - and GAD + cells projecting to OT (unpaired t-test: t5.598 = 2, p = 0.005). (d) Mean DF/F0 signal ±4s around sniffing enclosure initiation (indicated by dashed line, 0s) during habituation. (e) Mean DF/F0 signal ±4s around rearing initiation (indicated by dashed line, 0s) during habituation. (f) Mean DF/F0 signal ±4s around grooming initiation (indicated by dashed line, 0s) during habituation. (g) Quantification of DF/F0 difference before and after sniffing enclosure (grey), rearing (black) and grooming (red) start for the different phases of the test (Left: paired t-test, t1.932 = 5, p = 0.1113. Middle: paired t-test t3.163 = 5, p = 0.0250. Right: paired t-test, t4.703 = 5, p = 0.0053). (h) Mean DF/F0 signal ±4s around sniffing enclosure initiation (indicated by dashed line, 0s) during 1st exposure to CD1. (i) Mean DF/F0 signal ±4s around rearing initiation (indicated by dashed line, 0s) during 1st exposure to CD1. (j) Mean DF/F0 signal ±4s around grooming initiation (indicated by dashed line, 0s) during 1st exposure to CD1. (k) Quantification of DF/F0 difference before and after sniffing enclosure (blue), rearing (black) and grooming (red) start for the different phases of the test (Left: paired t-test, t2.826 = 5, p = 0.0369. Middle: paired t-test t4.002 = 5, p = 0.0103. Right: paired t-test, t7.568 = 5, p = 0.0006). (l) Mean DF/F0 signal ±4s around freezing initiation (indicated by dashed line, 0s) during threat recall. (m) Mean DF/F0 signal ±4s around rearing initiation (indicated by dashed line, 0s) during threat recall. (n) Mean DF/F0 signal ±4s around grooming initiation (indicated by dashed line, 0s) during threat recall. (o) Quantification of DF/F0 difference before and after freezing (purple), rearing (black) and grooming (red) start for the different phases of the test (Left: paired t-test, t0.8689 = 5, p = 0.4247. Middle: paired t-test t2.090 = 5, p = 0.0909. Right: paired t-test, t4.967 = 5, p = 0.0042).

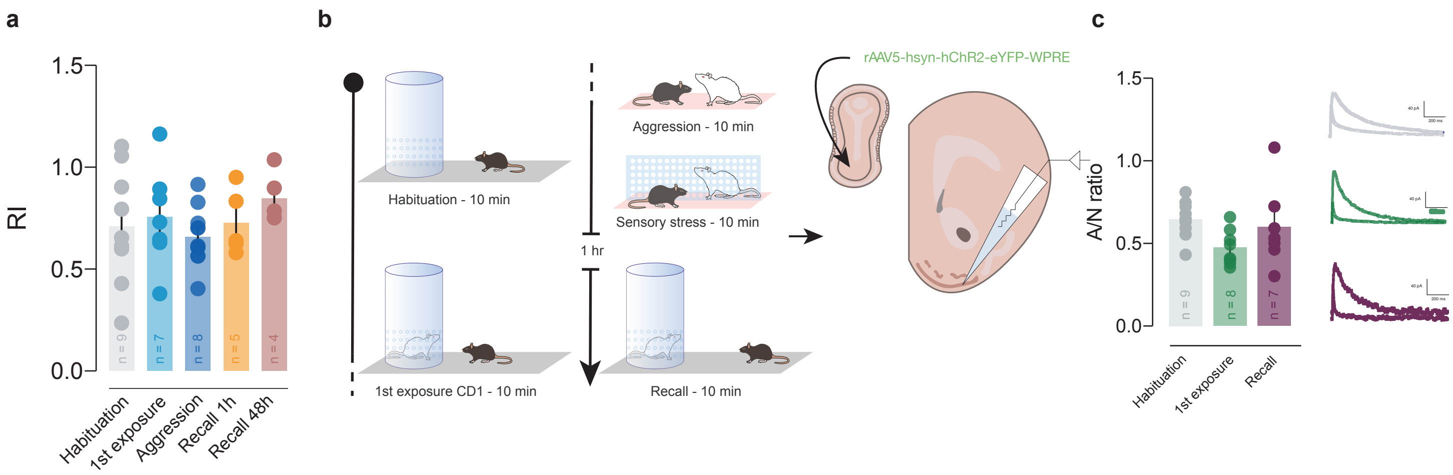

Supplementary Figure 5. (a) Bar graph representing rectification index (RI) recorded after different phases of the test (Ordinary one-way ANOVA: F(4, 31) = 0.6817. p = 0.6099). (b) Schematic of the experimental timeline and viral injection. (c) Left: Bar graph representing AMPAR/NMDAR ratio recorded after different phases of the test (Ordinary one-way ANOVA: F(2, 21) = 2.472. p = 0.1086). Right: Representative traces of AMPAR/NMDAR currents.

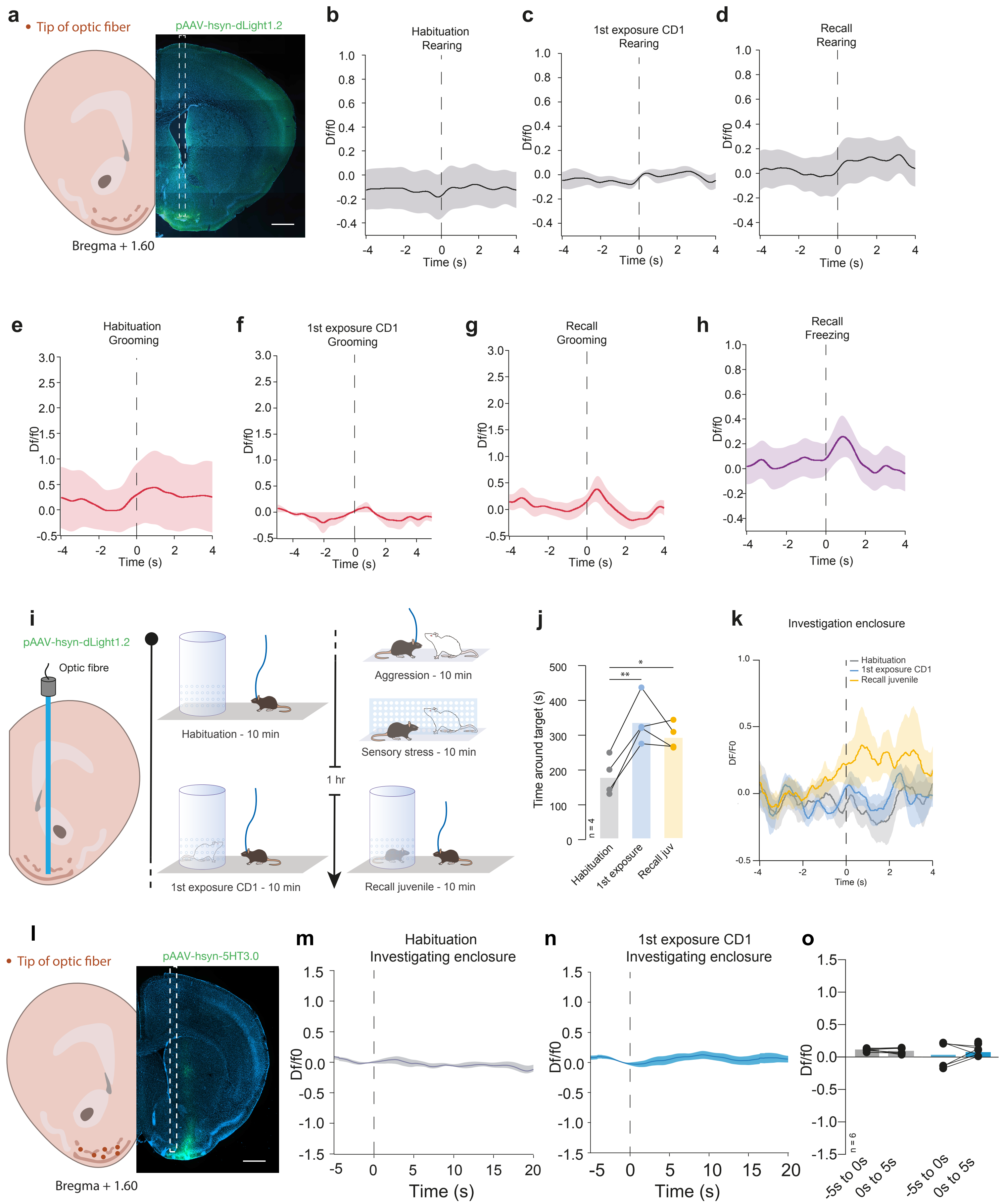

Supplementary Fig. 6, Casarotto et al

Supplementary Figure 6. (a) Optic fibers' tips localization after post-hoc validation and representative picture of injection and implantation side (scale bar: 500 µm). (b) Mean DF/F0 signal ±4s around rearing initiation (indicated by dashed line, 0s) during habituation. (c) Mean DF/F0 signal ±4s around rearing initiation (indicated by dashed line, 0s) during 1st exposure to CD1. (d) Mean DF/F0 signal ±4s around rearing initiation (indicated by dashed line, 0s) during recall. (e) Mean DF/F0 signal ±4s around grooming initiation (indicated by dashed line, 0s) during habituation. (f) Mean DF/F0 signal ±4s around grooming initiation (indicated by dashed line, 0s) during 1st exposure to CD1. (g) Mean DF/F0 signal ±4s around grooming initiation (indicated by dashed line, 0s) during recall. (h) Mean DF/F0 signal ±4s around freezing initiation (indicated by dashed line, 0s) during recall. (i) Left: Schematic of the viral injection. Right: Schematic of the experimental timeline. (j) Time around target (RM one-way ANOVA: $F_{1.178, 3.535} = 23.35$, $p = 0.0108$, followed by Bonferroni multiple comparison post hoc test). (k) Mean DF/F0 signal ±4s around investigating enclosure initiation (indicated by dashed line, 0s) during habituation, 1st exposure to CD1 and recall with juvenile. (l) Optic fibers' tips localization after post-hoc validation and representative picture of injection and implantation side (scale bar: 500 µm). (m) Mean DF/F0 signal ±4s around investigating enclosure initiation (indicated by dashed line, 0s) during habituation. (n) Mean DF/F0 signal ±4s around investigating enclosure initiation (indicated by dashed line, 0s) during 1st exposure to CD1. (o) Left: Quantification of DF/F0 difference before and after investigation the enclosure initiation for the habituation phase (paired t-test, $t_{1.181} = 5$, $p = 0.2908$). Quantification of DF/F0 difference before and after investigating the enclosure initiation for the 1st exposure to CD1 (paired t-test, $t_{0.5973} = 5$, $p = 0.5764$).

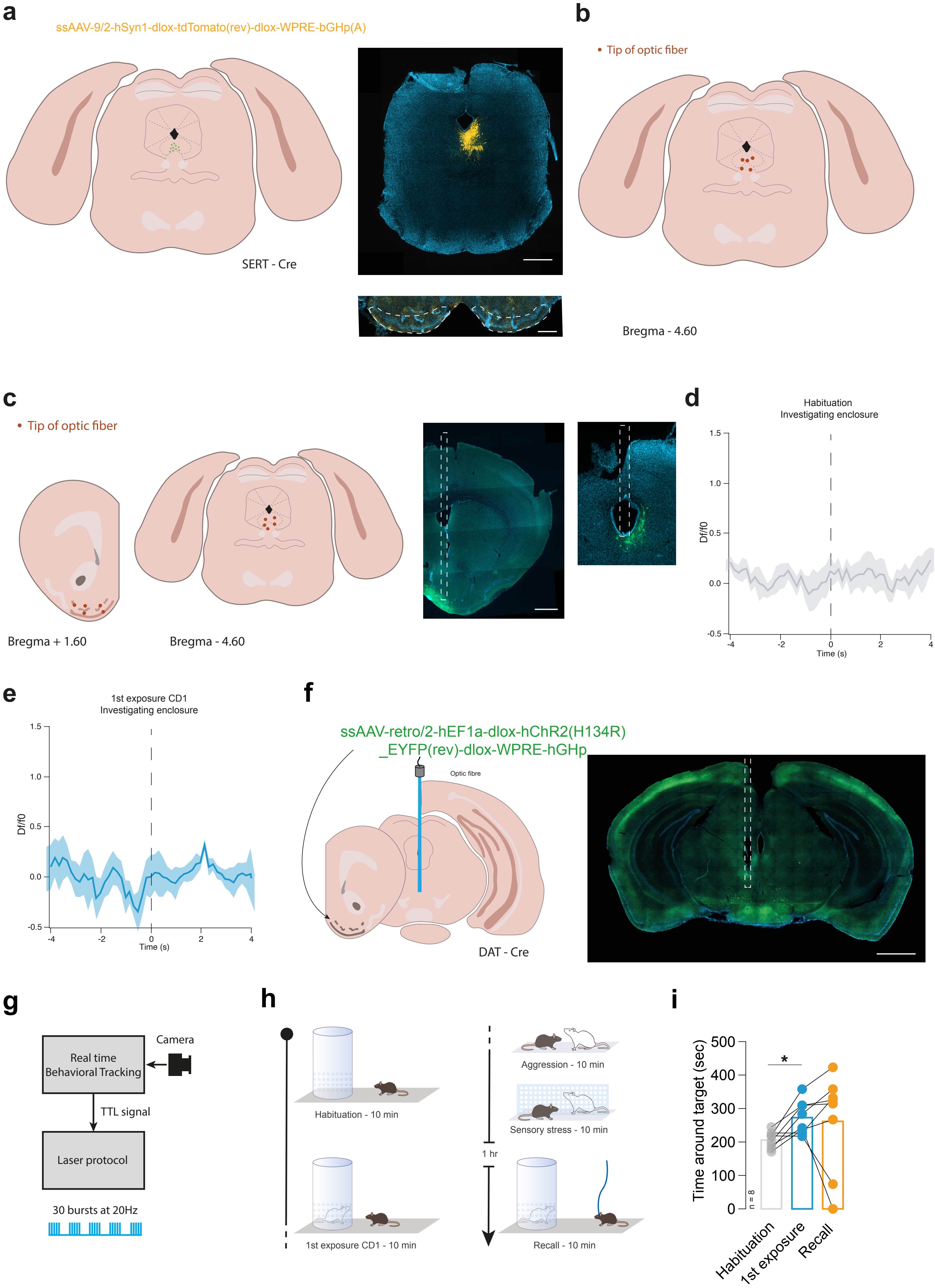

Supplementary Fig. 7, Casarotto et al

Supplementary Figure 7. (a) Left: Schematic of the viral injection. Right: Representative pictures of injection site (above) and fibers in the OT (below) in SERT-Cre mice (scale bar above: 750 µm, scale bar below: 500 µm). (b) Optic fibers's tips localization after post-hoc validation. (c) Left: Optic fibers's tips localization after post-hoc validation. Right: Representative pictures of viral injections and optic fibers implantation (scale bar: 750 µm). (d) Mean DF/F0 signal ±4s around investigating enclosure initiation (indicated by dashed line, 0s) during habituation. (d) Mean DF/F0 signal ±4s around investigating enclosure initiation (indicated by dashed line, 0s) during 1st exposure to CD1. (f) Left: Left: Schematic of the viral injection and optic fibers implantation. Right: Representative pictures of implantation site in the VTA (scale bar: 1000 µm). (g) Schematic of the closed loop stimulation system. (h) Schematic of the experimental timeline. (i) Time around target (RM one-way ANOVA: $F_{1.158, 8.105} = 1.628$, $p = 0.2423$, followed by Bonferroni multiple comparison post hoc test).
