# Supplementary material for: Olfactory tubercle mediates adaptive social behavior by controlling threat assessment and the expression of social threat memories during recall in male mice
Source: Nat Commun. 2026 May 19;17:6597. doi: 10.1038/s41467-026-73268-w (PMC13381864; doi:10.1038/s41467-026-73268-w)
Supplement: Supplementary file 2 — Reporting Summary [file 41467_2026_73268_MOESM2_ESM.pdf]

Corresponding author(s): Camilla Bellone

Last updated by author(s): Apr 2, 2026

Reporting Summary

Nature Portfolio wishes to improve the reproducibility of the work that we publish. This form provides structure for consistency and transparency in reporting. For further information on Nature Portfolio policies, see our Editorial Policies and the Editorial Policy Checklist.

Statistics

For all statistical analyses, confirm that the following items are present in the figure legend, table legend, main text, or Methods section.

n/a	Confirmed	
☐	☒	The exact sample size (*n*) for each experimental group/condition, given as a discrete number and unit of measurement
☐	☒	A statement on whether measurements were taken from distinct samples or whether the same sample was measured repeatedly
☐	☒	The statistical test(s) used AND whether they are one- or two-sided<br>*Only common tests should be described solely by name; describe more complex techniques in the Methods section.*
☒	☐	A description of all covariates tested
☐	☒	A description of any assumptions or corrections, such as tests of normality and adjustment for multiple comparisons
☐	☒	A full description of the statistical parameters including central tendency (e.g. means) or other basic estimates (e.g. regression coefficient) AND variation (e.g. standard deviation) or associated estimates of uncertainty (e.g. confidence intervals)
☐	☒	For null hypothesis testing, the test statistic (e.g. *F*, *t*, *r*) with confidence intervals, effect sizes, degrees of freedom and *P* value noted<br>*Give P values as exact values whenever suitable.*
☒	☐	For Bayesian analysis, information on the choice of priors and Markov chain Monte Carlo settings
☒	☐	For hierarchical and complex designs, identification of the appropriate level for tests and full reporting of outcomes
☒	☐	Estimates of effect sizes (e.g. Cohen's *d*, Pearson's *r*), indicating how they were calculated

Our web collection on statistics for biologists contains articles on many of the points above.

Software and code

Policy information about availability of computer code

Data collection	Ethovision XT 17.0, Noldus<br>Doric System fiber photometry, Doric Lenses inc.<br>Microscope Zeiss Software LSM800<br>Axioscan.Z1 Scanner Software<br>Igor Pro 6.34A
Data analysis	Ethovision XT 17.0, Noldus<br>Igor Pro 8.04<br>GraphPad Prism 10 (San Diego, CA, USA<br>Python 3.9<br>ZEN microscopy software, Zeiss

For manuscripts utilizing custom algorithms or software that are central to the research but not yet described in published literature, software must be made available to editors and reviewers. We strongly encourage code deposition in a community repository (e.g. GitHub). See the Nature Portfolio guidelines for submitting code & software for further information.

Data

Policy information about availability of data

All manuscripts must include a data availability statement. This statement should provide the following information, where applicable:

- Accession codes, unique identifiers, or web links for publicly available datasets
- A description of any restrictions on data availability
- For clinical datasets or third party data, please ensure that the statement adheres to our policy

Original data used in the present study are available in the following link: https://doi.org/10.5281/zenodo.19352359. Further data supporting the findings are available upon request. Find all the Source Data files attached to the manuscript. Each data file is provided for each figure and supplementary figure.

Research involving human participants, their data, or biological material

Policy information about studies with human participants or human data. See also policy information about sex, gender (identity/presentation), and sexual orientation and race, ethnicity and racism.

Reporting on sex and gender	n/a
Reporting on race, ethnicity, or other socially relevant groupings	n/a
Population characteristics	n/a
Recruitment	n/a
Ethics oversight	n/a

Note that full information on the approval of the study protocol must also be provided in the manuscript.

Field-specific reporting

Please select the one below that is the best fit for your research. If you are not sure, read the appropriate sections before making your selection.

[x] Life sciences [ ] Behavioural & social sciences [ ] Ecological, evolutionary & environmental sciences

For a reference copy of the document with all sections, see nature.com/documents/nr-reporting-summary-flat.pdf

Life sciences study design

All studies must disclose on these points even when the disclosure is negative.

Sample size	No statistical methods were used to predetermine the number of animals and cells. Suitable sample sizes were based on previous publications from the lab and are similar to those generally employed in the field.
Data exclusions	Statistical outliers were identified by using the criterion Mean(Value) +- 3 x std(value). However, in our study, no outliers have been identified using this criterion.
Replication	All behavioral experiments were replicate with success in different batches. Mean and SD did not vary significantly across experimental cohorths tested in separate occasions.
Randomization	All the stimuli mice, CD1 or juvenile stimuli, were randomly assigned to the experimental mice. All experimental mice were randomly assigned to either control or experimental conditions (e.g. chemo and optogenetic experiments).
Blinding	Experimenters were blinded to group allocation during data collection and analysis.

Reporting for specific materials, systems and methods

We require information from authors about some types of materials, experimental systems and methods used in many studies. Here, indicate whether each material, system or method listed is relevant to your study. If you are not sure if a list item applies to your research, read the appropriate section before selecting a response.

Materials & experimental systems

n/a	Involved in the study
[ ]	[x] Antibodies
[x]	[ ] Eukaryotic cell lines
[x]	[ ] Palaeontology and archaeology
[ ]	[x] Animals and other organisms
[x]	[ ] Clinical data
[x]	[ ] Dual use research of concern
[x]	[ ] Plants

Methods

n/a	Involved in the study
[x]	[ ] ChIP-seq
[x]	[ ] Flow cytometry
[x]	[ ] MRI-based neuroimaging

Antibodies

Antibodies used	Primary: rabbit anti-GFP (A-11122, Thermo Fisher Scientific), rat anti-tdTomato (16D7, Kerafast). Secondary: goat anti-rabbit IgG H&L (Alexa Fluor® 488, ThermoFisher, A11008), donkey anti-rat IgG H&L (Alexa Fluor® 555, ThermoFisher, A32794).
Validation	Primary and secondary antibodies were validated by the suppliers. Validation and relevant literature citations are available in the Thermo Fisher scientific and Kerafast website.<br><br>https://www.thermofisher.com/antibody/product/GFP-Antibody-Polyclonal/A-11122<br>https://www.kerafast.com/item/803/anti-tdtomato-16d7-antibody<br>https://www.thermofisher.com/antibody/product/Goat-anti-Rabbit-IgG-H-L-Cross-Adsorbed-Secondary-Antibody-Polyclonal/A-11008<br>https://www.thermofisher.com/antibody/product/Donkey-anti-Rabbit-IgG-H-L-Highly-Cross-Adsorbed-Secondary-Antibody-Polyclonal/A32794

Animals and other research organisms

Policy information about studies involving animals; ARRIVE guidelines recommended for reporting animal research, and Sex and Gender in Research

Laboratory animals	Adult (8-16 weeks) WT (C57BL/6J background), CD1, Fos-Cre ERt2, GAD-cre, DAT-cre and SERT-cre male mice were maintained on a 12h light-dark cycle (light on at 0700h). Mice had ad libitum access to food and water. The ambient temperature in the animal facility was 23°C and relative humidity around 50%.
Wild animals	No wild animals were used in this study
Reporting on sex	Experiments were conducted in male mice.
Field-collected samples	No field collected samples were used in this study
Ethics oversight	All procedures performed at UNIGE complied with the Swiss National Institutional Guidelines on Animal Experimentation and were approved by the respective Swiss Cantonal Veterinary Office Committees for Animal Experimentation.

Note that full information on the approval of the study protocol must also be provided in the manuscript.

Plants

Seed stocks	n/a
Novel plant genotypes	n/a
Authentication	n/a
