# Supplementary material for: Olfactory tubercle mediates adaptive social behavior by controlling threat assessment and the expression of social threat memories during recall in male mice
Source: Nat Commun. 2026 May 19;17:6597. doi: 10.1038/s41467-026-73268-w (PMC13381864; doi:10.1038/s41467-026-73268-w)
Supplement: Supplementary file 3 — Transparent Peer Review file [file 41467_2026_73268_MOESM3_ESM.pdf]

nature portfolio

Peer Review File

Olfactory Tubercle mediates adaptive social behavior by controlling threat assessment and the expression of social threat memories during recall in male mice.

Corresponding Author: Professor Camilla Bellone

This file contains all reviewer reports in order by version, followed by all author rebuttals in order by version.

Version 0:

Reviewer comments:

Reviewer #1

(Remarks to the Author)
In this manuscript, the authors applied a novel acute social threat assessment task and utilized a combination of viral tracing, chemogenetic and optogenetic inhibition, and slice electrophysiology to demonstrate connectivity to the olfactory tubercle (OT) that contributes to social threat assessment and responding. They focus on connectivity from the amygdala, though later incorporate neuromodulatory inputs from the raphe and ventral tegmentum. While other work detailed in the introduction indicates that the OT is important for olfactory and emotional behaviors, there remains little known about how the OT contributes to social behaviors (with exception to sociosexual behaviors as the authors cite).

It is interesting, as the authors report, that the OT appears to play a role in mediating threat assessment and threat memory – this entirely new for the role of the OT. The pathway-specific plasticity from the BLA to the OT is also previously unknown (although the authors approach doesn't completely document that, see below). Additionally, the differential effects of BLA to OT inhibition on passive and active defensive behaviors (freezing versus avoidance) provides a unique insight into a possible role of this circuit, which a semi-recent preprint has found is largely made up of DA receptor expressing neurons (see Sniffen & Ryu et al., 2024, https://doi.org/10.1101/2024.06.19.599749). Similarly, the finding that BLA input to the OT is active during grooming behavior is intriguing…though not really a focus of this and we're not sure we are on board with how that activity is indicative of some type of negative affective state since grooming can also be appetitive (just ask yourself if you like to scratch itches?).

These strengths stated, and again, while we are enthusiastic about the concept here, there are issues with conclusions and interpretations, and some missing experimental pieces to flesh this out completely. In multiple places there are logical leaps in key conclusions which are not supported by the current results (including conclusions claiming causality which were truly just phenomenological, conclusions about specific transmitters playing roles yet the only conclusion that can be made is that some brain regions may play roles, and also conclusions about specificity of BLA input to OT as mediating this threat behavior memory yet no attempt to rule out influence of BLA to accumbens input serving possibly redundant (or not) roles since BLA neurons send collaterals throughout the ventral striatum). These need to be better developed by new experiments and conclusions updated accordingly.

Major Comments:
1. The authors need to better convince that the outcomes is specific to BLA OT or is this also influencing BLA output to NAc —or is this influencing both? – in which case the authors should refer to this pathway more broadly as ventral striatum. This concern should be addressed in two ways – 1) At the minimum the authors need to provide more representative injection images and also maps for where expression was detected across all experimental animals.… especially for the DREADDs and GCamp7s, and 2) either a) based upon those outcomes break apart data from mice which had spill-out into the NAc or solely injections in the NAc and compare those to those with only confirmed injections in the TuS or b) inject mice into the NAc and OT separately and show if only the OT or NAc is necessary for defensive behaviors, or if both are necessary.
2. The proposed dopamine block on serotonergic signaling in the OT and the role of dopamine in directing social behaviors comes out of nowhere and is disjointed from the main story. Interesting, but not cohesive. Also, this experiment brings with it interpretability issues which require new experiments to answer. Viz., we need to know if the behaviors observed are really due to the actions of DA and 5HT on OT cells. Simply manipulating pathways from raphe or ventral tegmentum by no means

(as I'm sure the authors know) indicates a role for the 'canonical transmitters' from those regions. The authors need to rule out other transmitters and their possible co-release are not driving effects. The authors should include representative virus injection sites for all viral manipulations along with fiber tip locations for all fiber photometry experiments. Use of either pharmacologic antagonism upon optogenetic excitation would also provide further insight into the mechanism mediating this response.
3. Issue with slice data interpretation – is it long term plasticity or 'short term adaptation' as the authors argue? Slices are collected 24hours post behavior – seems on the long-term side. Also, the conclusion of these results read causally "threat recall INDUCES synaptic plasticity at BLA to OT synapses". How did the authors establish this causality? Do the A/N ration results when correlate with individual animal behavior outcomes? Group effects are provided but correlations are needed… and importantly… to prove that no correlation exists in the other groups of mice. It is possible the plasticity is evident in the other groups of mice which would more suggest this is a by product of some more general influence.
4. The specificity of the behaviors monitored are not well defined. The authors could use modern higher resolution analyses of behavioral strategies to define freezing, grooming, darting, investigation (currently referred to as sniffing), and avoidance. The current version of the manuscript does not necessarily demonstrate both passive (freezing) and active (darting) defensive behaviors since avoidance could also be a passive response correlating with increased freezing. Additionally, the rationale for defining freezing as 2 seconds of inactivity is not clear. Also, all references to sniffing should be updated with the term investigation since respiration was not measured so how can the authors conclude the mice are sniffing…they very well are seeking tactile input from vibrissae so "investigation" is more appropriate.
5. How do the authors reconcile the lack of dopamine signaling in the OT with recent literature demonstrating the role of dopamine in directing sniffing (Johnson et al., 2025, Nat Comm) and grooming (Zhang et al., 2023, Nat Comm)?
6. The timing of the DREADD mediated inhibition of the OT may not allow the authors to separate out the role of the OT in consolidation over retrieval, given the time course of i.p. injected CNO and the times at which the recall session was conducted. It would be interesting to see these results extended to the 24-hour time point like with the extinction training.
7. The task as it is currently described and represented in figures does not make it clear whether the experimental mouse and the CD1 conspecific are unable to interact each other. The raises questions about the authors claim that the threat is "stimulus specific". The 3mm diameter holes described could be large enough for a mouse's snout to poke through, and the purpose of the additional plexiglass barrier described is unclear. Our primary reason for concern here is that the mice could engage in "threatening" behaviors, such as dominance sniffing and ultrasonic vocalizations, but the authors have defined these CD1 mice as initially "non-threatening", though a better term may be "restrained". Additionally, the 10-minute period in the CD1 mouse homecage to increase "sensory stress" is omitted from the figure representations of the task, though it is a critical part of the novel task.
Minor Comments:
1. The authors should include n's in all figures or in figure legends, divided by sex.
2. Virus catalog numbers and sources should be included in the methods section.
3. Several references are repeated multiple times (8 and 10: Roy et al., 2022; 15 and 29: Gadziola et al., 2015) and citations in the Materials and Methods section are not included in References (e.g. Blanchard & Blanchard, 2003; Sturman et al., 2018...). Additionally, the 11th reference in the third paragram of the introduction should be the Gadziola et al., 2015 paper.
4. Further clarification is needed in how the df/f0 was calculated for all fiber photometry experiments. While we appreciated the use of appropriate control viruses for all optogenetic (Jaws vs EGFP) and DREADD (hM4D(Gi) vs mCherry) manipulations, the control (UV channel or mutant virus) for the fiber photometry signal (GCamp7s, dLight1.2, and 5HT3.0) is not referenced. Additionally, the rationale for averaging the 4 seconds prior to and after behavior is not clear. Does this match the typical length of the behaviors? Choosing these time frames may wash out behavior-related signals, so it may be helpful to see raster plots overlaid to demonstrate the average times at which the evaluated behaviors cease.
5. There are some recurring inconsistencies in the figures. Here are a few examples: Y axes in Fig 4: e.g. Freezing (s) for 4c, but Time freezing (s) in 4j, 400 s for upper freezing panels of Fig 4 but 600 s for lower freezing panels. Also, the CNO i.p. injection label is a very small arrow that looks like a stick in panel a until zooming in, even though it has a clear arrowhead in panel h. The n is indicated in Fig 2e, but nowhere else. A third-party person reviewing the figures for thematic consistency could be considered.
6. The authors note that when describing the results of Fig 5c that "the basolateral amygdala (BLA) exhibited the most robust activation among the identified regions" but did not show the quantification of Fos in the other regions (from the "CreERt2 mice cell counting" section in Methods): lateral olfactory tract (LOT), basolateral amygdala (BLA), paraventricular nucleus of the thalamus (PVT) and the anterior insular cortex (AIC)). This would be helpful for readers to see, especially since the neighboring piriform cortex, another primary olfactory structure involved in hedonics and odor processing, which sends monosynaptic input to the OT (https://doi.org/10.1523/jneurosci.1444-19.2019), also appears activated based on their BLA image (Fig 5c)
7. Fig 3 title says OT is "necessary" but the results with photometry are just phenomenological.

Reviewer #2

(Remarks to the Author)
In this manuscript Casarotto et al. investigate the role of the olfactory tubercle (OT) in recall of aversive social memories. Their major conclusion is that plasticity at the basolateral amygdala (BLA) to OT synapses is important in mediating the recall of threat memories and that 5-HT release into the OT facilitates avoidance and suppresses DA release which might control approach behavior. They conclude that the interaction of 5-HT and DA regulates the dynamic switch and updating in adaptive social behavior.

The major strength of this paper is that it not only uses functional circuit inhibition but also recording of cellular and neuromodulator activity along with synaptic plasticity recordings to understand the role of OT inputs and neuromodulators in

social threat memory recall. The findings are novel, the manuscript is well written, and the methods are appropriate. While I'm enthusiastic, there are some major and minor points below that need to be addressed.

Major:
- The stimulus mice used did not "become aggressive" as stated in the text. Rather the authors first restricted the access of the aggressors to the subject through a barrier, which is not an "ecologically valid context". The text needs to be rephrased, or a new paradigm could back up those claims.
- Likewise, I'm not convinced about the term "memory updating" as the subjects adapted to a new context in which they encountered the aggressor rather than updating the social memory. Also here, this needs to be either addressed with experiments or rephrased the conclusions.
- Further it is unclear whether OT has a specific role in social threat assessment or whether it is involved in general threat assessment as this was not tested with matching control experiments. Can the authors test whether the OT or BLA/DR inputs are specific for social threat assessment?
- BLA-OT neurons are active during sniffing of the enclosure as well as grooming. These are two very different behaviors. I wonder whether these neurons play a different broader role than threat memory and grooming, maybe attention?
- The authors inhibit the DR to OT inputs; however, 5-HT have been shown to co-release other neurotransmitters (PMID: 30146164). Therefore, the experiment does not conclusively show that 5-HT from the DR is important for the recall.
- The neuromodulatory switch only happened after artificial inhibition of 5-HT and thus it is unclear whether it would occur under natural conditions. Can the authors show that it also happens naturally e.g. after a change in social threat encounter?
- The authors cite (26) that the OT is densely innervated by serotonergic inputs from the DR. However, the cited paper did not discuss serotonergic inputs. Can the authors provide some more evidence that there are serotonergic inputs in the OT?
- It is not clear how the conclusions of the manuscript support the title that there is distinct control of threat assessment and memory updating.
- It is not discussed what the mechanisms of the 5-HT and DA interaction is on a physiological level.

Minor:
- From the flow it is not entirely clear why the authors shifted from exploring the role of BLA inputs to neuromodulation.
- It is surprising in Sup Fig 1b that the time around target is not less in the recall phase compared to 1st exposure. The assay used in Sup Fig 1b is similar to social memory tests performed by many labs (e.g. PMID: 24572357, PMID: 30301899). However, in this experiment the subjects seem to have impaired social memory. Does the exposure to the aggressor disrupt their performance in social memory assays with juveniles?
- In Figure 3, times -4 s to 0s is compared to 0 s to 4 s rather than comparing the peak z-scores between different conditions like most publications. A comparison between different conditions would be more robust as it shows neural activity specific to a behavior/experience. Can the authors compare the peak z-scores between e.g. habituation, 1st exposure and recall?
- The effect of CNO on DREADD activity usually lasts 6-10 h post injection (PMCID: PMC3758874), which means that in both 1st exposure (Fig. 4 a-g) and recall inhibition (Fig. 4 h-n) experiments, OT activity would be blunted during recall. However, the behavioral effect is only visible in the second experiment (Fig. 4 h-n). Can the authors explain the discrepancy? It would be better to use optogenetics to precisely time the inhibition.
- It is not apparent how many animals were used for Fig. 5c, which only shows 1 representative image per condition. Replicates are needed for this experiment, if the authors only used 1 animal.
- The authors show a decrease of AMPA/NMDA ratio at recall. Usually an increase in AMPA/NMDA suggests strengthening of the synapse during memory formation and a decrease indicates memory extinction. Can the authors explain how a decrease of AMPA/NMDA ratio mediates memory recall?
- In the discussion the authors speculate that the BLA-OT projection might regulate defensive behaviors. Can the authors explain which data supports this? It appears to me that the assays used in the manuscript only test for approach, avoidance and freezing.
- The number of animals used for each experiment is not indicated in the figure legends. Please add this information.
- Representative images of injection sites should be provided.
- The concentration of CNO for i.p. injections is not provided.
- It appears that the time around target is 2 cm in some experiments, but 5 cm in others. This discrepancy could influence behavior outcomes.

Reviewer #3

(Remarks to the Author)
In their manuscript, "Olfactory Tubercle mediates adaptive social behavior through distinct control of threat assessment and memory updating", Casarotto et al report a critical role of the olfactory tubercle (OT) in social threat assessment. The manuscript is beautifully structured. Experiments are elegant and methods are state-of-the-art. Data analyses are rigorous. Figures are super clear. The logical flow is outstanding. Findings are novel and exciting. This is a great paper as is. I only have a few minor comments.

1. Figure 3 shows Ca2+ transient analyses of OT neurons during behavior. These data do not demonstrate a causal role of identified activity in behavior (that is shown in Figure 4). Hence the title of Figure 3 needs to be corrected (necessity of OT activity is not assessed here).

2. Figure 4 shows that inhibition of the OT during recall abolishes avoidance behavior during recall. Yet, in the abstract the authors state that "inhibition [of OT activity during recall] prevented both the expression of avoidance behavior and subsequent memory updating". Also the title ascribes a role for the OT in "threat assessment and memory updating". I can't find the memory updating part (e.g. recall after 24 and 48 hours). Experiment(s) assessing memory subsequent to CNO

exposure should be added if the authors want to make a statement about both, expression of avoidance behavior during inhibition of the OT and effects on subsequent memory updating.

3. Figure 4 related results section. Freezing response seems to be diminished. Because there is no quantification of the degree and spread of chemogenetic OT inhibition, could it be that partial inhibition of the OT fully blocks avoidance behavior while only partially blocking freezing? In other words, I don’t think the data as presented can rule out a role for OT in freezing behavior.

4. Figure 5 related results section. not “transsynaptic” (e.g. rabies virus) but retrograde.

5. Figure 6 related results section. Instead of “Finally, to establish a causal link between BLA-OT activity…” better “Finally, to test for a causal link between BLA-OT activity…”

6. Figure 8 related results section. The viral/optogenetic inhibition approach does not target serotonergic neurons specifically but rather targets all neurons projecting from the DR to the OT. Hence the wording must be adjusted accordingly.

7. Figure 8 shows that Jaws-mediated inhibition of serotonergic (?) neurons projecting to the OT increases DA release in the OT during recall. It would be great if the authors could test if this “disinhibition” of DA release in the OT is driving approach behavior (see also below).

8. Re “This setup allowed us to simultaneously inhibit DR-OT 5-HT neurons in a closed-loop manner, as previously described”. Please describe closed-loop design here as this aspect is not clear.

9. Re “Together, these findings reveal a dynamic interplay between 5-HT and DA in the olfactory tubercle, where 5-HT release promotes social avoidance behavior, while its inhibition unmasks DA signaling that drives social approach, suggesting a crucial neuromodulatory balance in regulating adaptive social responses.” The authors have not shown that DA signaling drives social approach. This possibility remains to be tested.

10. In my opinion, the above comments can be addressed either experimentally, or alternatively by adjusting wording and conclusions.

11. The manuscript was a pleasure to read!

Version 1:

Reviewer comments:

Reviewer #1

(Remarks to the Author)
I think the authors have done a strong job of addressing our prior concerns. Lots of new analyses, data, and edits to the text have been included/made. While I think the circuit and neurochemical modulation (via DA and 5HT) may be more intricate than unveiled herein, I believe this manuscript is solid and a great contribution to the field.

Reviewer #2

(Remarks to the Author)
The authors have addressed all my concerns. I have no further comments.

Reviewer #3

(Remarks to the Author)
the authors have addressed my comments. i have nothing else to add.

Open Access This Peer Review File is licensed under a Creative Commons Attribution 4.0 International License, which permits use, sharing, adaptation, distribution and reproduction in any medium or format, as long as you give appropriate credit to the original author(s) and the source, provide a link to the Creative Commons license, and indicate if changes were made.
In cases where reviewers are anonymous, credit should be given to 'Anonymous Referee' and the source.
The images or other third party material in this Peer Review File are included in the article's Creative Commons license, unless indicated otherwise in a credit line to the material. If material is not included in the article's Creative Commons license and your intended use is not permitted by statutory regulation or exceeds the permitted use, you will need to obtain permission directly from the copyright holder.
To view a copy of this license, visit https://creativecommons.org/licenses/by/4.0/

REVIEWER COMMENTS

Reviewer #1 (Remarks to the Author):

In this manuscript, the authors applied a novel acute social threat assessment task and utilized a combination of viral tracing, chemogenetic and optogenetic inhibition, and slice electrophysiology to demonstrate connectivity to the olfactory tubercle (OT) that contributes to social threat assessment and responding. They focus on connectivity from the amygdala, though later incorporate neuromodulatory inputs from the raphe and ventral tegmentum. While other work detailed in the introduction indicates that the OT is important for olfactory and emotional behaviors, there remains little known about how the OT contributes to social behaviors (with exception to sociosexual behaviors as the authors cite).

It is interesting, as the authors report, that the OT appears to play a role in mediating threat assessment and threat memory – this entirely new for the role of the OT. The pathway-specific plasticity from the BLA to the OT is also previously unknown (although the authors approach doesn't completely document that, see below). Additionally, the differential effects of BLA to OT inhibition on passive and active defensive behaviors (freezing versus avoidance) provides a unique insight into a possible role of this circuit, which a semi-recent preprint has found is largely made up of DA receptor expressing neurons (see Sniffen & Ryu et al., 2024, https://doi.org/10.1101/2024.06.19.599749). Similarly, the finding that BLA input to the OT is active during grooming behavior is intriguing…though not really a focus of this and we're not sure we are on board with how that activity is indicative of some type of negative affective state since grooming can also be appetitive (just ask yourself if you like to scratch itches?).

These strengths stated, and again, while we are enthusiastic about the concept here, there are issues with conclusions and interpretations, and some missing experimental pieces to flesh this out completely. In multiple places there are logical leaps in key conclusions which are not supported by the current results (including conclusions claiming causality which were truly just phenomenological, conclusions about specific transmitters playing roles yet the only conclusion that can be made is that some brain regions may play roles, and also conclusions about specificity of BLA input to OT as mediating this threat behavior memory yet no attempt to rule out influence of BLA to accumbens input serving possibly redundant (or not) roles since BLA neurons send collaterals throughout the ventral striatum). These need to be better developed by new experiments and conclusions updated accordingly.

We thank the reviewers for identifying the novelty of our study and for the valuable comments and suggestions for improving the manuscript.

Major Comments:
1. The authors need to better convince that the outcomes is specific to BLA◊ OT or is this also influencing BLA output to NAc—or is this influencing both? – in which case the authors should refer to this pathway more broadly as ventral striatum. This concern should be addressed in two ways – 1) At the minimum the authors need to provide more representative injection images and also maps for where expression was detected across all experimental animals.… especially for the DREADDs and GCamp7s, and 2) either a) based upon those outcomes break apart data from mice which had spill-out into the NAc or solely injections in the NAc and compare those to those with only confirmed injections in the TuS or b) inject mice into the NAc and OT separately and show if only the OT or NAc is necessary for defensive behaviors, or if both are necessary.

We appreciate the reviewer's suggestion to clarify whether the observed effects are specific to the BLA→OT pathway or if they also involve the BLA→NAc pathway. To directly address this point, we performed additional experiments using inhibitory DREADDs specifically targeting the NAc. We found that inhibition of the NAc during recall did not prevent the avoidance behavior (new Supplementary Fig. 3f-g). This indicates that, at least within the odor-based defensive task we designed, the NAc is not necessary for the expression of this defensive behavior. Thus, the effects we observed can confidently be attributed to the BLA→OT pathway, rather than a broader effect involving the ventral striatum more generally. We have discuss this experiment and possible role of BLA to ventral striatum more generally in the discussion. In addition, as requested by the reviewer, we have included more detailed and representative images of injection sites across the entire manuscript. These additional controls further support the specificity of our manipulations to the OT rather than the NAc.

2. The proposed dopamine block on serotonergic signaling in the OT and the role of dopamine in directing social behaviors comes out of nowhere and is disjointed from the main story. Interesting, but not cohesive. Also, this experiment brings with it interpretability issues which require new experiments to answer. Viz., we need to know if the behaviors observed are really due to the actions of DA and 5HT on OT cells. Simply manipulating pathways from raphe or ventral tegmentum by no means (as I'm sure the authors know) indicates a role for the 'canonical transmitters' from those regions. The authors need to rule out other transmitters and their possible

co-release are not driving effects. The authors should include representative virus injection sites for all viral manipulations along with fiber tip locations for all fiber photometry experiments. Use of either pharmacologic antagonism upon optogenetic excitation would also provide further insight into the mechanism mediating this response.

We thank the reviewer for highlighting the need to clarify the relationship between neuromodulatory signaling (dopamine and serotonin) and synaptic plasticity mechanisms within our study.

We fully agree that dopamine and serotonin signals represent the acute mechanisms regulating immediate behavioral responses (approach or avoidance) during threat recall. Indeed, our data clearly show that serotonin release within the OT specifically facilitates acute avoidance behavior, while its inhibition unmasks dopamine release that promotes acute approach behavior. Thus, dopamine and serotonin dynamics serve as immediate neuromodulatory "switches" controlling adaptive social decisions at the moment of threat assessment. In contrast, the synaptic plasticity observed in the BLA→OT pathway emerges as a separate, subsequent event, contributing primarily to memory formation and consolidation processes. This plasticity is induced by recall experiences and persists even after extinction learning, providing a stable synaptic substrate for memory updating rather than directly mediating acute behavioral responses. We have now introduced the paragraph in the text as "Having established that the OT is required during recall and that BLA→OT inputs undergo recall-dependent plasticity, we next sought to identify the neuromodulatory mechanisms that regulate OT output during threat assessment. Because synaptic plasticity provides a substrate for storing updated threat information while neuromodulators shape moment-to-moment behavioral responses, we reasoned that monoaminergic systems might act on the OT to gate the expression of avoidance or approach following memory retrieval. In particular, dopamine and serotonin—both of which densely innervate the OT—are strong candidates to dynamically tune OT processing during recall. We therefore examined how DA and 5-HT signals interact with the OT circuitry during social threat evaluation."

To specifically address the reviewer's concern about potential co-release from raphe and VTA projections, we conducted additional control experiments. By injecting ChR2 and Chrimson into the raphe and VTA respectively, we tested for potential glutamatergic co-release within the OT. The absence of excitatory postsynaptic currents (EPSCs) following both the 488nm and 647nm light pulses indicates that these projections do not provide functional glutamatergic input to the sampled OT neurons. Here you can find the result of this experiment for the reviewer.

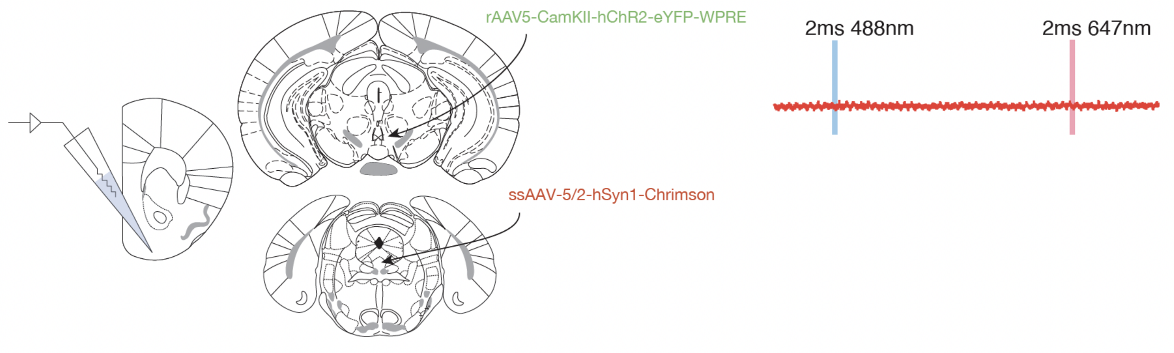

Fig A. Left: Schematic representation of viral vector injections and patch-clamp recording. Right: Optical stimulation protocol and representative trace. Brief 2ms pulses of 488nm (blue) and 647nm (red) light were applied to selectively activate ChR2 and Chrimson, respectively, in presence of PTX during electrophysiological monitoring to test for potential glutamate co-release.

We appreciate the reviewer's suggestion regarding pharmacological antagonism combined with optogenetic stimulation to further confirm the involvement of dopamine and serotonin. While we agree that these experiments would provide valuable additional evidence, their implementation poses significant technical challenges. Specifically, performing precise pharmacological manipulations would require cannula implantation directly into the OT to ensure localized drug delivery. Given the small size and anatomical complexity of the OT, accurately targeting and reliably maintaining these cannula placements is particularly challenging. Nevertheless, we conducted alternative control experiments to substantiate our interpretation. Specifically, we performed additional behavioral experiments using a juvenile (non-threatening) mouse instead of the aggressive CD1 mouse. In these

experiments, we observed an increase in dopamine signaling in the OT, accompanied by robust approach behavior—mirroring the dopamine dynamics we observed when serotonin signaling was inhibited during threat recall (Supplementary Fig. 6i–k). These results provide strong independent support for our interpretation that dopamine release in the OT promotes approach behaviors, further reducing the necessity of direct pharmacological manipulation. We have now clarified these technical limitations and included the juvenile control experiment in the manuscript, strengthening the interpretation and addressing the reviewer's concern about interpretability.

3. Issue with slice data interpretation – is it long term plasticity or 'short term adaptation' as the authors argue? Slices are collected 24hours post behavior – seems on the long-term side. Also, the conclusion of these results read causally "threat recall INDUCES synaptic plasticity at BLA to OT synapses". How did the authors establish this causality? Do the A/N ration results when correlate with individual animal behavior outcomes? Group effects are provided but correlations are needed…and importantly… to prove that no correlation exists in the other groups of mice. It is possible the plasticity is evident in the other groups of mice which would more suggest this is a by product of some more general influence.

We thank the reviewer for this comment. We agree that the synaptic changes we observe 24 hours after recall are more appropriately described as long-lasting synaptic plasticity rather than "short-term adaptation". We have therefore revised the wording throughout the manuscript to refer to "long-lasting synaptic plasticity". Regarding causality and the potential contribution of more general influences, we have clarified and strengthened the evidence in three ways:

Long-lasting, pathway- and phase-specific plasticity

As now emphasized in the Results, the decrease in AMPA/NMDA ratio at BLA→OT synapses displays a clear temporal and pathway specificity. First, this change is measured 24 hours after the recall session, consistent with a long-lasting form of synaptic plasticity rather than a transient adaptation. Second, it is absent in all other behavioral groups that underwent different phases of the paradigm (habituation, post-aggression without recall, extinction), indicating that it does not arise from exposure to stress or the task per se. Third, when we examined another major input to the OT, the olfactory bulb (OB), we did not detect comparable changes in synaptic transmission at OB→OT synapses (Supplementary Fig. 5b,c). Together, these observations argue against a non-specific by-product of stress or general task exposure and instead support a recall- and pathway-specific form of synaptic plasticity at BLA→OT synapses.

Correlation analyses with individual behavior

In line with the reviewer's suggestion, we have now systematically examined the relationship between synaptic plasticity and individual behavioral performance. For the recall group, we correlated the AMPA/NMDA ratio with the time spent in the target zone during recall. We did not observe a significant correlation, indicating that once recall-induced plasticity is established, its magnitude does not simply scale with the degree of avoidance behavior expressed by individual animals. This analysis is now presented in Fig. 6c (and detailed in the legend). We then performed the same correlation analyses for the behavioral group undergoing chemogenetic inhibition (Fig. 6d-e) and likewise did not detect significant relationships between AMPA/NMDA ratio and time spent in the target zone; these data are shown in Fig. 6f.

Establishing a causal link between recall-evoked OT activity, behavior, and plasticity

To directly address causality, we have the chemogenetic experiment (Fig. 6c–d). Chemogenetic inhibition of OT neurons (hM4Di) selectively during the recall session prevents both the expression of social avoidance behavior and the decrease in AMPA/NMDA ratio at BLA→OT synapses measured 24 hours later. Under these conditions, the AMPA/NMDA ratios remain comparable to those of control animals that do not show recall-associated plasticity. Thus, OT activity during recall is necessary for both the behavioral adaptation and the synaptic change, supporting the conclusion that recall-evoked activity within the BLA→OT pathway drives the observed plasticity, rather than reflecting a more general, recall-independent influence.

4. The specificity of the behaviors monitored are not well defined. The authors could use modern higher

resolution analyses of behavioral strategies to define freezing, grooming, darting, investigation (currently referred to as sniffing), and avoidance. The current version of the manuscript does not necessarily demonstrate both passive (freezing) and active (darting) defensive behaviors since avoidance could also be a passive response correlating with increased freezing. Additionally, the rationale for defining freezing as 2 seconds of inactivity is not clear. Also, all references to sniffing should be updated with the term investigation since respiration was not measured so how can the authors conclude the mice are sniffing…they very well are seeking tactile input from vibrissae so "investigation" is more appropriate.

We thank the reviewer for these constructive suggestions. We have clarified our behavioral definitions and expanded our analyses accordingly.

Behavioral definitions and terminology

We now explicitly define each scored behavior in the Methods (freezing, grooming, rearing, investigation of the enclosure, and avoidance).

As requested, we have replaced all references to "sniffing" with "investigation of the enclosure", since respiration was not measured and the animals could also rely on tactile input from the vibrissae. This change has been implemented throughout the manuscript.

Freezing is now clearly defined as ≥2 s of complete immobility (no locomotion, rearing, or grooming) except for respiratory movements. A 2-s threshold is widely used in the literature and excludes brief pauses in locomotion while capturing sustained defensive immobility; we now state this rationale in the Methods.

Passive vs active defensive strategies: transition probabilities

To better characterize how defensive strategies evolve across phases, we now quantify the transition probabilities between grooming, rearing, and investigation of the enclosure (and, during recall, freezing) (Supplementary Fig. 1a). This analysis reveals a clear shift in the structure of behavioral sequences at recall: transitions *into* investigation of the enclosure are reduced, while transitions *out of* investigation towards grooming or freezing are increased. These changes indicate that avoidance is not simply an increase in passive freezing, but reflects a reorganization of exploratory and defensive strategies specifically when the previously aggressive CD1 is encountered again. This is now represented in Supplementary Fig. 1a.

Spatial organization of avoidance

To further dissociate spatial avoidance from generalized freezing, we now provide **zone occupancy analyses**. We quantified the proportion of time spent in three zones: (i) around the target enclosure, (ii) in the center of the arena, and (iii) at the opposite end of the corridor. These data are presented as pie charts for the different sessions (Fig. 1c, d). During recall, mice spend significantly less time near the CD1 enclosure and proportionally more time at the far end of the corridor, consistent with an active spatial avoidance strategy rather than merely increased immobility in the vicinity of the threat.

On higher-resolution behavioral methods

We agree that modern, high-dimensional approaches (e.g. unsupervised clustering of pose data) are powerful tools to dissect fine-grained behavioral motifs. In the present study, however, our main goal was to relate well-defined, ethologically meaningful categories (freezing, spatial avoidance, investigation of the enclosure, grooming, rearing) to OT and BLA→OT activity. We therefore relied on these established measures, now complemented by transition-probability and zone-occupancy analyses that more precisely capture how defensive strategies are reorganized at recall. We acknowledge in the Discussion that future work using higher-resolution methods could further refine the ethogram of social threat responses.

5. How do the authors reconcile the lack of dopamine signaling in the OT with recent literature demonstrating the role of dopamine in directing sniffing (Johnson et al., 2025, Nat Comm) and grooming (Zhang et al., 2023, Nat Comm)?

We do not interpret our data as contradicting a role of dopamine in sniffing or grooming. In fact, we now show that DA transients are clearly detected in the OT when mice explore a novel juvenile conspecific after the CD1 experience, and when DR→OT neurons are inhibited during recall, approach to the CD1 enclosure is again accompanied by DA release (Fig. 8n; Supplementary Fig. 6i–k). Thus, OT DA is readily engaged in positive or disinhibited exploratory contexts, consistent with Johnson et al. and Zhang et al.

The key difference is the threat recall context and the specific behavior we measure. In our task, the mouse performs “investigation of the enclosure” through an opaque barrier, rather than direct nose-to-body sniffing. We also observe only small, brief DA peaks at grooming onset, which are not selectively enhanced during recall and do not show the robust, threat-locked pattern seen for 5-HT. Our data therefore suggest that during social threat recall, 5-HT signaling in the OT predominates and biases behavior toward avoidance, whereas DA-driven exploratory signals are largely suppressed and only re-emerge when 5-HT input is inhibited.

6. The timing of the DREADD mediated inhibition of the OT may not allow the authors to separate out the role of the OT in consolidation over retrieval, given the time course of i.p. injected CNO and the times at which the recall session was conducted. It would be interesting to see these results extended to the 24-hour time point like with the extinction training.

We thank the reviewer for this insightful comment regarding the timing of DREADD-mediated inhibition and its potential confounding effects on consolidation versus retrieval processes. The reviewer correctly identified that the pharmacokinetics of intraperitoneally injected CNO and the timing of our recall sessions could potentially complicate the interpretation of whether OT's role occurs during memory consolidation or retrieval phases.
In response to this concern, we conducted the additional experiments suggested by the reviewer. We extended our analysis to examine the effects of OT inhibition on memory recall at the 24-hour time point, similar to our extinction training paradigm. These experiments involved administering CNO to selectively inhibit OT activity specifically during the recall session 24hr after the experience.
We observed that animals still showed no avoidance behavior when tested 24 hours later. This finding demonstrates that OT's role in our behavioral paradigm is indeed critical during the retrieval phase rather than during consolidation. These data are now shown in Supplementary Fig. 3a-e.

7. The task as it is currently described and represented in figures does not make it clear whether the experimental mouse and the CD1 conspecific are unable to interact each other. The raises questions about the authors claim that the threat is “stimulus specific”. The 3mm diameter holes described could be large enough for a mouse’s snout to poke through, and the purpose of the additional plexiglass barrier described is unclear. Our primary reason for concern here is that the mice could engage in “threatening” behaviors, such as dominance sniffing and ultrasonic vocalizations, but the authors have defined these CD1 mice as initially “non-threatening”, though a better term may be “restrained”. Additionally, the 10-minute period in the CD1 mouse homecage to increase “sensory stress” is omitted from the figure representations of the task, though it is a critical part of the novel task.

In response to the reviewer's concerns about the interaction between the experimental mouse and the CD1 conspecific, we have clarified our experimental setup. The 3mm diameter holes in the barrier are indeed small enough to prevent direct physical contact between animals, as they are smaller than a CD1's snout diameter. However, the reviewer correctly points out that these holes do allow for limited sensory interaction, including olfactory and potentially acoustic communication. The additional plexiglass barrier serves as a secondary containment measure to ensure complete physical separation while still permitting the transmission of relevant sensory cues.
The reviewer makes an excellent point regarding our terminology. We agree that describing the CD1 mice as "non-threatening" is imprecise, given that restrained animals can still engage in threatening behaviors such as dominance-related olfactory signaling and ultrasonic vocalizations. We have revised our terminology throughout the manuscript to describe these animals as "restrained" rather than "non-threatening," which more accurately reflects their physical constraint rather than making assumptions about their behavioral state or signaling capacity.
We have also addressed the reviewer's concern about the omission of the 10-minute period in the CD1 mouse homecage from our figure representations. This sensory stress component is indeed a critical aspect of our novel task design, as it allows the experimental mouse to experience the full range of olfactory and acoustic

cues associated with the conspecific before the formal testing begins. We have now incorporated this component into our figure representations to provide a complete visual description of the experimental timeline and ensure that readers understand the full scope of sensory exposure in our paradigm.

Minor Comments:
1. The authors should include n's in all figures or in figure legends, divided by sex.
We thank the reviewer for this important methodological point. We have now included sample sizes (n's) for all experimental groups in our figures, either directly on the figures themselves or in the figure legends, with clear indication of how the samples are divided by sex.

2. Virus catalog numbers and sources should be included in the methods section.
We have now included the complete virus catalog numbers and sources for all viral constructs used in our study in the methods section.

3. Several references are repeated multiple times (8 and 10: Roy et al., 2022; 15 and 29: Gadziola et al., 2015) and citations in the Materials and Methods section are not included in References (e.g. Blanchard & Blanchard, 2003; Sturman et al., 2018...). Additionally, the 11th reference in the third paragram of the introduction should be the Gadziola et al., 2015 paper.

We thank the reviewer for identifying these formatting and citation errors. We will correct all duplicate references in the reference list, ensuring that Roy et al., 2022 and Gadziola et al., 2015 appear only once with consistent numbering throughout the manuscript. We will also add all missing citations from the Materials and Methods section (including Blanchard & Blanchard, 2003 and Sturman et al., 2018) to the References section. Additionally, we will correct the citation in the third paragraph of the introduction to properly reference Gadziola et al., 2015.

4. Further clarification is needed in how the df/f0 was calculated for all fiber photometry experiments. While we appreciated the use of appropriate control viruses for all optogenetic (Jaws vs EGFP) and DREADD (hM4D(Gi) vs mCherry) manipulations, the control (UV channel or mutant virus) for the fiber photometry signal (GCamp7s, dLight1.2, and 5HT3.0) is not referenced. Additionally, the rationale for averaging the 4 seconds prior to and after behavior is not clear. Does this match the typical length of the behaviors? Choosing these time frames may wash out behavior-related signals, so it may be helpful to see raster plots overlaid to demonstrate the average times at which the evaluated behaviors cease.

We thank the reviewer for these comments. We have updated the Methods section to clarify our data processing and behavioral filtering.

5. There are some recurring inconsistencies in the figures. Here are a few examples: Y axes in Fig 4: e.g. Freezing (s) for 4c, but Time freezing (s) in 4j, 400 s for upper freezing panels of Fig 4 but 600 s for lower freezing panels. Also, the CNO i.p. injection label is a very small arrow that looks like a stick in panel a until zooming in, even though it has a clear arrowhead in panel h. The n is indicated in Fig 2e, but nowhere else. A third-party person reviewing the figures for thematic consistency could be considered.

We thank the reviewer for these comments regarding the thematic consistency and clarity of our figures. We have corrected all identified inconsistencies by standardizing the Y-axis labels across the figures and making the illustrations more visible and clear. Furthermore, we have ensured the sample size (n) for each group is explicitly indicated in every figure legend.

6. The authors note that when describing the results of Fig 5c that "the basolateral amygdala (BLA) exhibited the most robust activation among the identified regions" but did not show the quantification of Fos in the other regions (from the "CreERt2 mice cell counting" section in Methods): lateral olfactory tract (LOT), basolateral amygdala (BLA), paraventricular nucleus of the thalamus (PVT) and the anterior insular cortex (AIC)). This would be helpful for readers to see, especially since the neighboring piriform cortex, another primary olfactory structure involved in hedonics and odor processing, which sends monosynaptic input to the OT (https://doi.org/10.1523/jneurosci.1444-19.2019), also appears activated based on their BLA image (Fig 5c)

We thank the reviewer for this suggestion. We have now added the quantification of cFos+ cells/mm² to Supplementary Fig. 4a, which confirms that the BLA is the region exhibiting the highest level of activation.

7. Fig 3 title says OT is "necessary" but the results with photometry are just phenomenological.

We will revise the title of Figure 3 to reflect this more accurately, removing the term "necessary"

Reviewer #2 (Remarks to the Author):

In this manuscript Casarotto et al. investigate the role of the olfactory tubercle (OT) in recall of aversive social memories. Their major conclusion is that plasticity at the basolateral amygdala (BLA) to OT synapses is important in mediating the recall of threat memories and that 5-HT release into the OT facilitates avoidance and suppresses DA release which might control approach behavior. They conclude that the interaction of 5-HT and DA regulates the dynamic switch and updating in adaptive social behavior.

The major strength of this paper is that it not only uses functional circuit inhibition but also recording of cellular and neuromodulator activity along with synaptic plasticity recordings to understand the role of OT inputs and neuromodulators in social threat memory recall. The findings are novel, the manuscript is well written, and the methods are appropriate. While I'm enthusiastic, there are some major and minor points below that need to be addressed.

Major:
- The stimulus mice used did not "become aggressive" as stated in the text. Rather the authors first restricted the access of the aggressors to the subject through a barrier, which is not an "ecologically valid context". The text needs to be rephrased, or a new paradigm could back up those claims.

We agree that our previous wording overstated the "ecological validity" of the paradigm. We have now rephrased the Results to describe the procedure more explicitly: mice first investigate a restrained CD1 through a perforated enclosure and then undergo a brief resident-intruder-like free interaction in the aggressor's home cage, followed by barrier-separated exposure. We now refer to this as a "controlled, ethologically inspired context" rather than claiming full ecological validity

- Likewise, I'm not convinced about the term "memory updating" as the subjects adapted to a new context in which they encountered the aggressor rather than updating the social memory. Also here, this needs to be either addressed with experiments or rephrased the conclusions.

We agree that our original wording may overstate what we directly demonstrate. Our experiments show recall-dependent changes in the expression of a social threat memory (avoidance vs approach), together with long-lasting, recall-gated synaptic plasticity in the BLA→OT pathway. However, we do not directly dissect the representational content of the memory trace itself.
To better reflect this, we have now revised the terminology throughout the manuscript. Specifically, we no longer claim "memory updating" as a primary conclusion and instead refer to "recall-dependent expression of social threat memories" and "long-lasting synaptic plasticity associated with changes in threat expression". We retain the term "memory updating" only when discussing theoretical frameworks in the Discussion, where we now explicitly state that our data are consistent with-rather than directly proving-an updating of the social threat memory. We have also change the title "Olfactory Tubercle mediates adaptive social behavior through distinct control of threat assessment and the expression of social threat memories."

- Further it is unclear whether OT has a specific role in social threat assessment or whether it is involved in general threat assessment as this was not tested with matching control experiments. Can the authors test whether the OT or BLA/DR inputs are specific for social threat assessment?

We thank the reviewer for this important question regarding the specificity of OT's role in social versus general threat assessment. To address this concern, we conducted additional control experiments using innately aversive stimuli. Specifically, we tested mice with trimethylthiazoline (TMT), a predator odor that elicits immediate avoidance responses. Our results show that while mice immediately avoided TMT as expected, inhibition of OT did not prevent this avoidance behavior. This finding demonstrates that OT's role is specific to social threat learning rather than being involved in general threat detection or innate avoidance responses.
We acknowledge that additional control experiments using footshock would be challenging to implement in our paradigm, as mice typically respond to footshock with freezing behavior rather than the active avoidance behaviors we measure in our social threat assessment task. This fundamental difference in behavioral response patterns would make it difficult to create a comparable experimental paradigm that could adequately test the specificity of OT function across different threat modalities. The TMT control experiments therefore provide the most appropriate comparison for assessing the specificity of OT's role in our behavioral paradigm, demonstrating that OT function is selectively required for learned social threat responses but not for immediate responses to innate non-social threats. The new data are now in Supplementary Fig. 3 h-j.

- BLA-OT neurons are active during sniffing of the enclosure as well as grooming. These are two very different

behaviors. I wonder whether these neurons play a different broader role than threat memory and grooming, maybe attention?

We agree with the reviewer that BLA→OT neurons are unlikely to be exclusively dedicated to threat memory. As shown in our event-aligned analyses, these neurons are activated also during non-threat-related behaviors such as grooming across phases.. This pattern is consistent with a broader role in encoding the salience or attentional selection of behaviorally relevant social events, rather than a pure “threat memory” signal. We have revised the Discussion to explicitly propose that BLA→OT projections may convey a more general salience/attention-related signal to the OT, which is then integrated with serotonergic and dopaminergic inputs to shape the expression of social threat memories and grooming.

- The authors inhibit the DR to OT inputs; however, 5-HT have been shown to co-release other neurotransmitters (PMID: 30146164). Therefore, the experiment does not conclusively show that 5-HT from the DR is important for the recall.

We thank the reviewer for this important concern regarding potential co-release of other neurotransmitters from DR neurons, as documented in the cited literature (PMID: 30146164). To specifically address this concern about potential co-release from raphe projections, we conducted additional control experiments using optogenetic approaches. By injecting Crimson into the raphe and optogenetically stimulating these terminals within the OT, we tested for potential glutamatergic co-release and found no evidence of glutamate release from these inputs.
These control experiments presented here for the reviewer provide crucial evidence that our observed effects are specifically attributable to serotonergic signaling rather than co-released neurotransmitters.

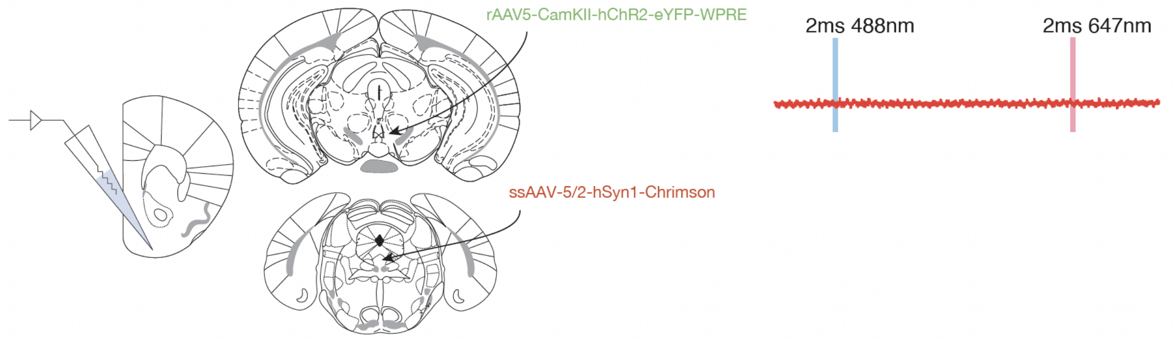

Fig A. Left: Schematic representation of viral vector injections and patch-clamp recording. Right: Optical stimulation protocol and representative trace. Brief 2ms pulses of 488nm (blue) and 647nm (red) light were applied to selectively activate ChR2 and Chrimson, respectively, in presence of PTX during electrophysiological monitoring to test for potential glutamate co-release.

- The neuromodulatory switch only happened after artificial inhibition of 5-HT and thus it is unclear whether it would occur under natural conditions. Can the authors show that it also happens naturally e.g. after a change in social threat encounter?

We thank the reviewer for this important point. In the original version, the most explicit “switch” between 5-HT and DA indeed came from optogenetic inhibition of DR→OT 5-HT neurons. In the revised manuscript, we now provide additional evidence that OT dopamine release is naturally engaged under non-threatening social conditions.

Specifically, when mice interact with a juvenile conspecific, we observe DA transients in the OT during recall of this non-aggressive social encounter (Supplementary Fig. 6i–k), in contrast to the absence of DA phasic release during recall of the aggressive adult CD1. Thus, within the same overall behavioral framework, OT neuromodulation differs as a function of social threat level: 5-HT is recruited during recall of a previously aggressive adult, whereas DA is engaged during recall of a positive juvenile interaction, without any experimental manipulation of 5-HT. Together, the juvenile-conspecific data and the DR→OT inhibition experiments support the view that a natural,

context-dependent neuromodulatory shift between 5-HT–dominated and DA–dominated OT signaling can occur, and that our manipulation reveals the underlying causal contribution of 5-HT to suppressing DA-driven approach during social threat recall.

- The authors cite (26) that the OT is densely innervated by serotonergic inputs from the DR. However, the cited paper did not discuss serotonergic inputs. Can the authors provide some more evidence that there are serotonergic inputs in the OT?

We thank the reviewer for this comment and agree that in the paper it is not clearly stated that the OT is innervated by serotoninergic fibers. To answer this comment, we have injected an anterograde cre dependent virus expressing a reporter protein in the Dorsal Raphe of SERT cre mice. We then sliced the brains and check for 5-HT fibers in the OT (Sup. Fig. 7a).

- It is not clear how the conclusions of the manuscript support the title that there is distinct control of threat assessment and memory updating.

We thank the reviewer for this comment and agree that our original wording was too strong. We have now revised both the title and the text to better match what we directly demonstrate. The title is not "Olfactory Tubercle mediates adaptive social behavior by controlling threat assessment and the expression of social threat memories."

- It is not discussed what the mechanisms of the 5-HT and DA interaction is on a physiological level.

We thank the reviewer for this comment. We have added the following paragraph in the discussion "At a physiological level, our data are consistent with a model in which DR-derived 5-HT constrains DA-dependent approach signals in the OT through a combination of pre- and postsynaptic mechanisms. Serotonin receptors expressed on OT medium spiny neurons are well positioned to modulate their excitability and integration of dopaminergic input, for example by biasing the relative recruitment of D1- and D2-type populations and thereby shifting the balance between approach and avoidance states. In addition, 5-HT receptors on presynaptic terminals of DA afferents, or on local interneurons, could directly regulate DA release probability and/or the gain of DA-sensitive synapses. In this framework, strong DR→OT activation during social threat recall would both enhance the responsiveness of avoidance-promoting OT ensembles and suppress DA-driven approach signals, whereas inhibition of DR→OT input would release this brake, allowing DA transients to emerge during approach. Although our experiments do not identify the specific receptor subtypes or cell classes involved, they provide a circuit-level constraint that future work can use to dissect the precise synaptic and molecular interactions between 5-HT and DA in the OT.

Minor:
- From the flow it is not entirely clear why the authors shifted from exploring the role of BLA inputs to neuromodulation.

We thank the reviewer for this helpful comment. To clarify the logical transition between the circuit-level analysis and the neuromodulatory experiments, we have added an explicit bridging paragraph at the beginning of the section *"DA and 5-HT balance in social approach/avoidance behavior"*: "Having established that the OT is required during recall and that BLA→OT inputs undergo recall-dependent plasticity, we next sought to identify the neuromodulatory mechanisms that regulate OT output during threat assessment. Because synaptic plasticity provides a substrate for storing updated threat information while neuromodulators shape moment-to-moment behavioral responses, we reasoned that monoaminergic systems might act on the OT to gate the expression of avoidance or approach following memory retrieval. In particular, dopamine and serotonin—both of which densely innervate the OT—are strong candidates to dynamically tune OT processing during recall. We therefore examined how DA and 5-HT signals interact with the OT circuitry during social threat evaluation."

This paragraph makes explicit that, after identifying BLA→OT plasticity as a candidate substrate for recall-dependent changes, we next asked how monoaminergic inputs modulate OT output on faster timescales to control the expression of social avoidance versus approach.

- It is surprising in Sup Fig 1b that the time around target is not less in the recall phase compared to 1st exposure. The assay used in Sup Fig 1b is similar to social memory tests performed by many labs (e.g. PMID: 24572357, PMID: 30301899). However, in this experiment the subjects seem to have impaired social memory. Does the exposure to the aggressor disrupt their performance in social memory assays with juveniles?

To address the reviewer's hypothesis that exposure to the aggressor might disrupt performance in social memory assays with juveniles, we conducted additional control experiments. We tested whether prior aggression exposure would generalize to affect social interactions with juvenile conspecifics. Our results show that mice exposed to our social threat paradigm maintain normal investigative behavior toward juvenile mice, indicating that the learned avoidance response is stimulus-specific and does not generalize to other social contexts. These data are now represented in Supplementary Fig. 6 i-j. This finding demonstrates that our social threat paradigm does not impair general social approach behaviors or create a broad social avoidance phenotype, but rather produces selective learned responses to the specific threatening stimulus. The preservation of normal social behavior toward juveniles suggests that the underlying social recognition and approach mechanisms remain intact following our experimental manipulation.

- In Figure 3, times -4 s to 0s is compared to 0 s to 4 s rather than comparing the peak z-scores between different conditions like most publications. A comparison between different conditions would be more robust as it shows neural activity specific to a behavior/experience. Can the authors compare the peak z-scores between e.g. habituation, 1st exposure and recall?

We thank the reviewer for this suggestion. We have addressed this by comparing the DF/F0 across experimental conditions to highlight the neural activity specific to the behavioral experience. Our results demonstrate that the peak DF/F0 during the recall session is significantly higher than that observed during both habituation and the initial exposure, confirming an enhancement in neural representation during this phase.

- The effect of CNO on DREADD activity usually lasts 6-10 h post injection (PMCID: PMC3758874), which means that in both 1st exposure (Fig. 4 a-g) and recall inhibition (Fig. 4 h-n) experiments, OT activity would be blunted during recall. However, the behavioral effect is only visible in the second experiment (Fig. 4 h-n). Can the authors explain the discrepancy? It would be better to use optogenetics to precisely time the inhibition.

We thank the reviewer for this comment. In the "aggression inhibition" cohort (Fig. 4a–g), CNO was injected 30 min before the first exposure/aggression block, and recall took place ~1.5–2 h later. Thus, OT activity is likely reduced both during the initial aggressive encounter and, to a lesser extent, during recall. The key observation in this cohort is that despite this early inhibition (and possible residual inhibition at recall), mice still show robust avoidance when re-exposed to the aggressor.

In contrast, in the "recall inhibition" cohort (Fig. 4h–n), CNO was administered 30 min before the recall session, such that OT inhibition is maximal at the time when avoidance normally emerges. Under these conditions, the difference in interaction time between first exposure and recall is abolished, while freezing is spared. We further replicate this effect in an independent cohort in which recall is tested 24 h after aggression and CNO is given only before recall (Supplementary Fig. 3a–e), ruling out lingering effects from the initial aggression day.

Taken together, these experiments indicate that strong OT inhibition at recall reliably prevents the expression of learned avoidance, whereas OT inhibition centred on the initial aggressive encounter (with only residual effect at recall) does not.

- It is not apparent how many animals were used for Fig. 5c, which only shows 1 representative image per condition. Replicates are needed for this experiment, if the authors only used 1 animal.

We thank the reviewer for this comment. We have updated the figure legends and the methods section to explicitly state the number of animals used for these experiments. We confirm that multiple replicates were used for each condition and have now added the corresponding quantification in Supplementary Fig. 4a.

- The authors show a decrease of AMPA/NMDA ratio at recall. Usually an increase in AMPA/NMDA suggests strengthening of the synapse during memory formation and a decrease indicates memory extinction. Can the authors explain how a decrease of AMPA/NMDA ratio mediates memory recall?

We thank the reviewer for this important question regarding the decrease in AMPA/NMDA ratio during recall. The reviewer correctly notes that increased AMPA/NMDA ratios are typically associated with synaptic strengthening during memory formation, while decreases are often linked to extinction processes.
Our observed decrease in AMPA/NMDA ratio during recall may reflect a distinct mechanism from traditional memory consolidation or extinction paradigms. Rather than indicating memory weakening, this decrease could represent a form of synaptic homeostasis or metaplasticity that occurs during memory retrieval. The reduction in AMPA/NMDA ratio might facilitate the flexibility needed for memory updating and behavioral adaptation during recall, allowing the circuit to integrate new contextual information with existing memories.

- In the discussion the authors speculate that the BLA-OT projection might regulate defensive behaviors. Can the authors explain which data supports this? It appears to me that the assays used in the manuscript only test for approach, avoidance and freezing.

We thank the reviewer for this comment and agree that our wording was too broad. We have revised the opening of the Discussion accordingly. We now state that the OT mediates “social threat assessment and the expression of social threat–related approach and avoidance behaviors” instead of “defensive behavior,”.

- The number of animals used for each experiment is not indicated in the figure legends. Please add this information.

We thank the reviewer for this comment. We have updated the figure legends to explicitly state the number of animals used for each experiment.

- Representative images of injection sites should be provided.

We thank the reviewer for this comment. We have now included more detailed and representative images of injection sites across the entire manuscript.

- The concentration of CNO for i.p. injections is not provided.

We thank the reviewer for this observation. We have updated the Methods section to include the specific concentration and dosage of CNO used for all i.p. injections (3 mg/kg). This information has also been added to the relevant figure legends to ensure clarity.

- It appears that the time around target is 2 cm in some experiments, but 5 cm in others. This discrepancy could influence behavior outcomes.

We thank the reviewer for pointing out the apparent discrepancy in the distance thresholds used. We have clarified this in the Methods section. More specifically, we have standardized all offline analysis using a 2 cm threshold. However, for the online closed-loop experiments, we intentionally set the activation threshold to 5 cm. This decision was made because the closed-loop stimulation relies on real-time tracking from Ethovision, which can occasionally swap the "nose" and "tail" point assignments. While these tracking errors are easily corrected during post-hoc video analysis, they cannot be corrected during the live stimulation. By using a 5 cm zone, we ensure that the laser triggers when the body center is within the target area, which effectively guarantees that the nose is within the relevant 2 cm zone even if a point-swap occurs. This approach provides a more robust and reliable trigger for the closed-loop system during the experiment.

Reviewer #3 (Remarks to the Author):

In their manuscript, “Olfactory Tubercle mediates adaptive social behavior through distinct control of threat assessment and memory updating”, Casarotto et al report a critical role of the olfactory tubercle (OT) in social threat assessment. The manuscript is beautifully structured. Experiments are elegant and methods are state-of-the-art. Data analyses are rigorous. Figures are super clear. The logical flow is outstanding. Findings are novel and exciting. This is a great paper as is. I only have a few minor comments.

1. Figure 3 shows Ca2+ transient analyses of OT neurons during behavior. These data do not demonstrate a causal role of identified activity in behavior (that is shown in Figure 4). Hence the title of Figure 3 needs to be corrected (necessity of OT activity is not assessed here).

We thank the reviewer for this correction. We agree that Figure 3 describes the activity of the OT and does not demonstrate causality, which is addressed via the manipulations in Figure 4. Accordingly, we have changed the title of Figure 3 to "OT neurons exhibit increased activity during social threat assessment." to more accurately reflect the data showing higher neural activation during this specific task phase compared to others.

2. Figure 4 shows that inhibition of the OT during recall abolishes avoidance behavior during recall. Yet, in the abstract the authors state that "inhibition [of OT activity during recall] prevented both the expression of avoidance behavior and subsequent memory updating". Also the title ascribes a role for the OT in "threat assessment and memory updating". I can't find the memory updating part (e.g. recall after 24 and 48 hours). Experiment(s) assessing memory subsequent to CNO exposure should be added if the authors want to make a statement about both, expression of avoidance behavior during inhibition of the OT and effects on subsequent memory updating.

We thank the reviewer for this helpful comment. We agree that in the original version we did not directly assess memory after OT inhibition. In the revised manuscript, we have now added an additional cohort in which recall is tested 24 h after the aggression experience, with CNO given 30 min before this 24 h recall session. In this cohort, control mice show robust avoidance at 24 h, whereas OT inhibition by CNO prevents this avoidance response (Supplementary Fig. 3a–e). Together with the 1 h data, this shows that OT activity is required for the expression of social avoidance at both early and later recall time points.

However, we still do not re-test the same animals after CNO washout. We therefore no longer claim that OT inhibition affects "subsequent memory updating" and have removed this wording from the abstract and title. We now restrict our conclusion to a role of the OT in controlling the expression of social threat memories during recall.

3. Figure 4 related results section. Freezing response seems to be diminished. Because there is no quantification of the degree and spread of chemogenetic OT inhibition, could it be that partial inhibition of the OT fully blocks avoidance behavior while only partially blocking freezing? In other words, I don't think the data as presented can rule out a role for OT in freezing behavior.

We thank the reviewer for this important point. In Figure 4, freezing during recall in the hM4Di+CNO group shows a slight reduction in magnitude, but this difference does not reach statistical significance when compared to controls. Thus, our data demonstrate a robust and specific loss of social avoidance, whereas effects on freezing are at most partial and not clearly resolved with our current approach.

We agree that, in the absence of a direct quantification of the degree and spatial extent of OT inhibition, we cannot rule out a contribution of OT to freezing behavior. We have therefore softened our interpretation in the Results and Discussion: rather than stating that the OT is "not essential" for freezing, we now conclude that OT activity is critical for the expression of learned social avoidance, while our data do not exclude a role in modulating freezing. We also explicitly acknowledge this limitation and note that future work using temporally precise, spatially defined inhibition (e.g. optogenetics) will be required to fully clarify the OT's contribution to freezing responses.

4. Figure 5 related results section. not "transsynaptic" (e.g. rabies virus) but retrograde.

We thank the reviewer for this correction regarding the viral tracing terminology. We have updated the Results section related to Figure 5 to replace the term "transsynaptic" with "retrograde" to accurately reflect the nature of the viral labeling used in these experiments.

5. Figure 6 related results section. Instead of "Finally, to establish a causal link between BLA-OT activity…" better "Finally, to test for a causal link between BLA-OT activity…"

We thank the reviewer for this suggestion. We agree that the revised wording is more precise, and we have rephrased the text in the Results section accordingly.

6. Figure 8 related results section. The viral/optogenetic inhibition approach does not target serotonergic neurons specifically but rather targets all neurons projecting from the DR to the OT. Hence the wording must be

adjusted accordingly.

We thank the reviewer for this important clarification regarding the specificity of our optogenetic targeting. We have adjusted the wording in the Results section for Fig. 8 to accurately reflect that our approach targets the population of neurons projecting from the DR to the OT. Specifically, we have replaced references to "serotonergic neurons" with "putative serotonergic neurons" or "DR-OT projecting neurons" throughout the text to acknowledge the potential presence of non-serotonergic projections within this circuit.

7. Figure 8 shows that Jaws-mediated inhibition of serotonergic (?) neurons projecting to the OT increases DA release in the OT during recall. It would be great if the authors could test if this “disinhibition” of DA release in the OT is driving approach behavior (see also below).

We thank the reviewer for this suggestion. To investigate whether the increase in DA release influences behavior, we performed an experiment where we "disinhibited" DA release via optogenetic stimulation of VTA DA neurons projecting to the OT, using a DAT-Cre mouse line. These results are now included in Supplementary Fig. 7f-i.

Specifically, we utilized a real-time closed-loop system where behavioral tracking triggered laser stimulation whenever the animal entered the target zone during the recall phase. Our findings show that stimulation of the VTA-OT projection did not result in avoidance; instead, animals continued to spend significant time around the target. These data support the functional relevance of DA release modulation in this circuit.

8. Re “This setup allowed us to simultaneously inhibit DR-OT 5-HT neurons in a closed-loop manner, as previously described”. Please describe closed-loop design here as this aspect is not clear.

We thank the reviewer for this request for clarification. We have updated the manuscript to provide a more detailed description of the closed-loop design used for the real-time inhibition of DR-OT neurons.

To implement the closed-loop inhibition, we used a real-time behavioral tracking system (Ethovision XT 17.0 (Noldus, Wageningen, the Netherlands) to monitor the animal's position relative to the enclosure. Specifically, we defined a virtual circular "target zone" centered on the enclosure with a diameter 5 cm larger than the enclosure itself (øzone=øenclosure+5 cm). Whenever the tracking software detected the animal within this zone, it triggered a TTL pulse that initiated continuous 640 nm laser delivery for the entire duration of the stay. We chose a 5 cm threshold for the online trigger to ensure high reliability; the 5 cm buffer for the online trigger accounts for potential tracking "swaps" between the nose and tail points, ensuring that the laser is active whenever the animal's body center is in proximity to the enclosure. This setup ensures that even during a point-swap, the nose remains within the relevant 2 cm interaction area during stimulation.

9. Re “Together, these findings reveal a dynamic interplay between 5-HT and DA in the olfactory tubercle, where 5-HT release promotes social avoidance behavior, while its inhibition unmasks DA signaling that drives social approach, suggesting a crucial neuromodulatory balance in regulating adaptive social responses.” The authors have not shown that DA signaling drives social approach. This possibility remains to be tested.

We thank the reviewer for this point. To address whether DA signaling in the OT influences social behavior, we performed an experiment using a real-time closed-loop system where behavioral tracking triggered laser stimulation of VTA-DA neurons projecting to the OT whenever the animal entered the target zone. As shown in Supplementary Fig. 7i, stimulation of this VTA-OT projection did not promote avoidance. This result indicates that while 5-HT release promotes social avoidance, the unmasking of DA signaling promotes social approach, supporting our conclusion that a neuromodulatory balance in the OT regulates adaptive social responses.

10. In my opinion, the above comments can be addressed either experimentally, or alternatively by adjusting wording and conclusions.
We thank the reviewer for this constructive feedback and for the flexibility provided in addressing these concerns. We have taken a comprehensive approach to strengthening the manuscript by both performing additional experiments and carefully rephrasing our conclusions to align with the data.

As suggested, we have addressed the reviewers' comments through a combination of new experimental data and refined terminology:

- Experimental Additions: We performed new closed-loop optogenetic experiments stimulating the VTA-OT dopaminergic projection during threat recall. These data, presented in Supplementary Fig. 7f-i,

demonstrate that "disinhibiting" DA release does not promote avoidance but instead promotes social approach, as animals spent significantly more time around the target compared to habituation.

- Wording and Conclusion Adjustments:
    - We modified the title of Figure 3 to "OT neurons exhibit increased activity during social threat assessment" to avoid implying causality before it is demonstrated in Figure 4.
    - We removed claims regarding "memory updating" from the title and abstract, focusing instead on the expression of social threat memories.
    - We adopted more precise terminology, replacing "transsynaptic" with "retrograde" and referring to DR-OT projections as "putative serotonergic neurons".
- Technical Clarifications: We have detailed the closed-loop design in the Methods, explaining the use of a 5 cm virtual "target zone" to ensure reliable laser activation even during potential tracking swaps between the nose and tail points.

These changes ensure that our conclusions regarding the neuromodulatory balance between 5-HT and DA in the OT are robustly supported by both existing and new evidence.

11. The manuscript was a pleasure to read!
